# Targeting the Exonic Circular *OGT* RNA/O-GlcNAc Transferase/Forkhead Box C1 Axis Inhibits Asparagine- and Alanine-Mediated Ferroptosis Repression in Neuroblastoma Progression

**DOI:** 10.34133/research.0703

**Published:** 2025-05-23

**Authors:** Qilan Li, Yang Cheng, Chunhui Yang, Minxiu Tian, Xiaojing Wang, Dan Li, Xinyue Li, Jiaying Qu, Shunchen Zhou, Liduan Zheng, Qiangsong Tong

**Affiliations:** ^1^ Department of Pediatric Surgery, Union Hospital, Tongji Medical College, Huazhong University of Science and Technology, Wuhan 430022, Hubei Province, P. R. China.; ^2^ Department of Pathology, Union Hospital, Tongji Medical College, Huazhong University of Science and Technology, Wuhan 430022, Hubei Province, P. R. China.; ^3^ Department of Geriatrics, Union Hospital, Tongji Medical College, Huazhong University of Science and Technology, Wuhan 430022, Hubei Province, P. R. China.

## Abstract

The disruption of ferroptosis, an emerging form of programmed cell death, is crucial in the development and aggressiveness of tumors. Meanwhile, the mechanisms and treatments that control ferroptosis in neuroblastoma (NB), a prevalent extracranial cancer in children, are still unknown. In this study, forkhead box C1 (FOXC1) and O-GlcNAc transferase (OGT) are identified as regulators of asparagine- and alanine-mediated ferroptosis repression in NB. Mechanistically, OGT facilitates FOXC1 stabilization via inducing O-GlcNAcylation in liquid condensates to increase the expression of asparagine synthetase (*ASNS*) and glutamate pyruvate transaminase 2 (*GPT2*), resulting in asparagine and alanine biogenesis, and subsequent synthesis of cystathionine β-synthase (CBS) or ferritin heavy chain 1 (FTH1). Meanwhile, exonic circular *OGT* RNA (*ecircOGT*) is able to encode a novel protein (OGT-570aa) containing domain essential for binding of OGT to FOXC1, which competitively decreases the OGT–FOXC1 interaction. Preclinically, miconazole nitrate facilitates the interaction of OGT-570aa with FOXC1, suppresses ferroptosis resistance of NB cells, and inhibits their growth, invasion, and metastasis. In clinical NB cases, higher *OGT*, *FOXC1*, *ASNS*, *GPT2*, *CBS*, or *FTH1* levels are correlated with worse survival, while lower *ecircOGT* or OGT-570aa expression is associated with tumor progression. These results indicate that targeting the *ecircOGT*/*OGT*/*FOXC1* axis inhibits asparagine- and alanine-mediated ferroptosis repression in NB progression.

## Introduction

Neuroblastoma (NB), the most common extracranial solid malignancy in infants and young children, originates from aberrant neural crest cells and accounts for 10% to 15% of childhood cancer deaths [1]. Clinically, NB exhibits heterogeneous behaviors including spontaneous regression or rapid progression [1,2]. For high-risk NB patients, despite multimodal treatment such as surgical resection, radiotherapy, high-dose chemotherapy, stem cell transplantation, and immunotherapy, the long-term survival rate is less than 50%, mainly due to tumor relapse or metastasis [1]. Therefore, a deeper understanding of mechanisms underlying tumorigenesis and aggressiveness is critical for proposing novel strategy of NB treatment.

Ferroptosis is a type of cell death triggered by lipid reactive oxygen species (ROS) that relies on iron, and is distinct from apoptosis or necroptosis in terms of morphological features [3–6]. In ferroptotic cell death, iron combines with hydrogen peroxide to produce hydroxyl radicals via Fenton reaction [7], a process facilitated by autophagic breakdown of iron-storing ferritin heavy chain 1 (FTH1) [8]. Besides, amino acids modulate ferroptosis through ‌maintaining redox homeostasis [9]. Among them, cysteine is an essential precursor of glutathione (GSH) that is critical for detoxifying lipid peroxides via glutathione peroxidase 4 (GPX4)-mediated conversion to oxidized glutathione (GSSG) [10], while GSSG is transformed back to GSH by consuming nicotinamide adenine dinucleotide phosphate hydrogen (NADPH) [10]. As an important component of trans-sulfuration pathway, cystathionine β-synthase (CBS) converts homocysteine to cysteine [11] and mediates resistance to ferroptosis in liver [11], breast [12], or ovarian [13] cancer. *CBS* fosters the development and aggressiveness of colon cancer [14], whereas lower *CBS* levels are linked to glioma progression [15], indicating its oncogenic or tumor-suppressive functions depending on the context. Nevertheless, additional investigation is required to explore the molecular basis and treatment approaches that control ferroptosis in tumor progression.

Forkhead box C1 (FOXC1) belongs to the FOX transcription factor family featured by a winged helix DNA-binding domain [16] and participates in embryonic development, proliferation, or metabolism [16]. Abnormal expression of *FOXC1* is documented in liver [17], gastric [18], esophageal [19], or prostate [20] cancer, and is linked to unfavorable outcomes of patients [17]. As a transcription factor, FOXC1 promotes tumor invasion or metastasis by increasing the expression of neural precursor cell expressed developmentally down-regulated 9 (*NEDD9*) [17] or matrix metalloprotease 7 (*MMP7*) [21]. In hepatocellular carcinoma (HCC), *FOXC1* facilitates C-C motif chemokine ligand 2 (*CCL2*) or C-X-C motif chemokine receptor 1 (*CXCR1*) expression to drive tumor-associated macrophage infiltration and metastasis [22]. In addition, *FOXC1* regulates stem cell properties via activating smoothened-independent Hedgehog signaling in breast cancer [23]. The expression or activity of *FOXC1* is controlled at posttranslational levels [24,25]. Extracellular signal-regulated kinase 1/2 (ERK1/2) induces S272 phosphorylation and prolongs half-life of FOXC1 protein in cervical cancer [24], while SUMOylation of FOXC1 inhibits its transcriptional activity [25]. These findings indicate that *FOXC1* exerts oncogenic functions, while its targets and regulatory mechanisms in ferroptosis still need to be identified.

In this research, we discover that *FOXC1* enhances the expression of de novo asparagine or alanine biogenesis genes, asparagine synthetase (*ASNS*) and glutamate pyruvate transaminase 2 (*GPT2*), which are linked to poor prognosis of NB patients. Notably, amino acid deprivation triggers direct interaction of O-GlcNAc transferase (OGT) with FOXC1 in liquid condensates, resulting in O-GlcNAcylation and stabilization of FOXC1 to resist ferroptosis via increasing asparagine/alanine biogenesis and subsequent CBS or FTH1 expression. Meanwhile, a novel protein consisting of 570 amino acids (OGT-570aa) encoded by exonic circular *OGT* RNA (*ecircOGT*) is able to competitively bind with FOXC1, leading to a decrease in OGT–FOXC1 interaction, *ASNS* and *GPT2* expression, asparagine and alanine biogenesis, and ferroptosis resistance of NB cells. Preclinically, administration of miconazole nitrate (MN) facilitates the interaction of OGT-570aa with FOXC1, and inhibits the growth and severity of tumors, indicating the roles of the *ecircOGT*/*OGT*/*FOXC1* axis in asparagine/alanine biogenesis and ferroptosis resistance during NB progression.

## Results

### *FOXC1* facilitates asparagine and alanine biogenesis in tumor cells

**Fig. 1.**
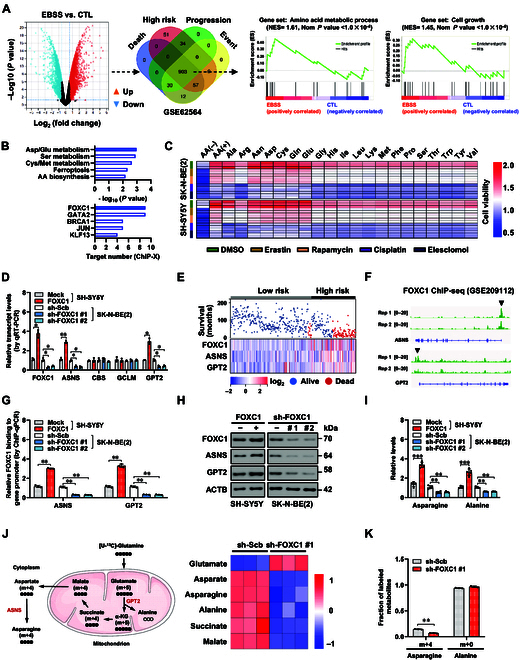
*FOXC1* facilitates asparagine and alanine biogenesis in NB cells. (A) Volcano plot (left panel) showing differentially expressed genes (fold change >2.0, *P* < 0.01) in SH-SY5Y cells treated with control (CTL) DMEM/F12 or EBSS for 6 h. Venn diagram (middle panel) and GSEA (right panels) revealing their association with death, high risk, clinical progression, and event in 498 NB cases (GSE62564), as well as biological functions. (B) KEGG pathway analysis (top panel) and ChIP-X program (bottom panel) revealing the involvement and transcriptional regulators of amino acid metabolic genes. (C) MTT colorimetric assay indicating the viabilities of SH-SY5Y cells treated with amino acids (AA), AA deprivation [AA(−)], or supplement of alanine (Ala), arginine (Arg), asparagine (Asn), aspartate (Asp), cysteine (Cys), glutamine (Gln), glutamate (Glu), glycine (Gly), histidine (His), isoleucine (Ile), leucine (Leu), lysine (Lys), methionine (Met), phenylalanine (Phe), proline (Pro), serine (Ser), threonine (Thr), tryptophan (Trp), tyrosine (Tyr), or valine (Val), and those treated with DMSO, erastin (5.0 μmol·l^−1^), rapamycin (1.0 μmol·l^−1^), cisplatin (5.0 μmol·l^−1^), or elesclomol (20 nmol·l^−1^) for 16 h. (D) Real-time qRT-PCR assay indicating the levels (normalized to *β-actin*, *n* = 5) of *FOXC1*, *ASNS*, *CBS*, *GCLM*, or *GPT2* in SH-SY5Y or SK-N-BE(2) cells stably transfected with empty vector (mock), *FOXC1*, scramble shRNA (sh-Scb), sh-FOXC1 #1, or sh-FOXC1 #2. (E) Heatmap indicating the expression profiles of *FOXC1*, *ASNS*, or *GPT2* in 498 NB cases (GSE62564) with different risk stratification, alive status, or survival time. (F) ChIP-seq peak (GSE209112) revealing endogenous enrichment of FOXC1 on promoter regions of *ASNS* and *GPT2* in HepG2 cells. (G and H) ChIP-qPCR (G, normalized to input, *n* = 3) and Western blot (H) assays revealing the FOXC1 enrichment or expression levels of *ASNS* and *GPT2* in SH-SY5Y or SK-N-BE(2) cells stably transfected with mock, *FOXC1*, sh-Scb, sh-FOXC1 #1, or sh-FOXC1 #2. (I) Relative asparagine and alanine levels in SH-SY5Y or SK-N-BE(2) cells stably transfected with mock, *FOXC1*, sh-Scb, sh-FOXC1 #1, or sh-FOXC1 #2 (*n* = 5). (J and K) Schematic illustration, heatmap (J), and quantification (K) of ^13^C-glutamine metabolic flux assay indicating the levels of metabolites within SK-N-BE(2) cells stably transfected with sh-Scb, or sh-FOXC1 #1 (*n* = 3). Fisher’s exact test for overlapping analysis in (A). Student’s *t* test or ANOVA compared the difference in (D), (G), (I), and (K). *P < 0.05, **P < 0.01, ***P < 0.001. Data are shown as mean ± SEM (error bars) or representative of 3 independent experiments in (D) to (K).

To investigate the mechanisms regulating tumorigenesis, RNA sequencing (RNA-seq) assay was performed to show 1,118 elevated and 1,392 reduced genes in SH-SY5Y, an established NB cell line model for neuronal response to nutrition deprivation [26,27], after treatment by Earle’s balanced salt solution (EBSS) free of amino acids (Fig. 1A). Through mining of a publicly available dataset of 498 NB patients (GSE62564), 903 genes were consistently linked to different stages of mortality, risk stratification, clinical advancement, and events (Fig. 1A). Gene set enrichment analysis (GSEA) showed obvious involvement of these genes in amino acid metabolism or cell growth with a normalized enrichment score (NES) of 1.61 (normalized *P* value < 1.0 × 10^−4^) or 1.45 (normalized *P* value < 1.0 × 10^−4^), especially in Asp/Glu, serine (Ser), or Cys/methionine (Met) metabolism (Fig. 1B). Analysis using the ChIP-X program [28] indicated top 5 transcriptional regulators of these amino acid metabolic genes, including FOXC1, GATA binding protein 2 (GATA2), breast cancer gene 1 (BRCA1), Jun proto-oncogene (JUN), and KLF transcription factor 13 (KLF13; Fig. 1B and Table S1). Further analysis of public datasets revealed that only *FOXC1* was consistently associated with poor survival of 498 (GSE62564), 283 (GSE85047), 102 (GSE3446), and 88 (GSE16476) NB patients (Fig. S1A). In cultured NB cells, supplements of alanine (Ala), asparagine (Asn), aspartate, cysteine, glutamine, or glutamate were able to resist cell death triggered by inducers of ferroptosis (erastin) or autophagy (rapamycin), but not by those of apoptosis (cisplatin) or cuproptosis (elesclomol, Fig. 1C). The activity, but not transcript levels, of *FOXC1* was enhanced by EBSS treatment (Fig. S1B and C). Western blot assay showed the increase of FOXC1 levels within cultured NB cell lines, prostate cancer PC-3 cells, and cervical cancer HeLa cells, when compared to those in embryonic kidney HEK293 cells (Fig. S1D). Based on endogenous FOXC1 levels, SH-SY5Y, SK-N-AS, PC-3 (exhibiting relatively low expression), SK-N-BE(2), IMR-32, and HeLa (with relatively high expression) cell lines were selected for further investigation (Fig. S1D). Steady overexpression or silencing of *FOXC1* resulted in higher or lower levels of *ASNS* or *GPT2* transcripts, without alteration in those of *CBS* or glutamate-cysteine ligase modifier subunit (*GCLM*, Fig. 1D). In 498 NB patients (GSE62564), the levels of *FOXC1*, *ASNS*, or *GPT2* were linked to risk stratification, survival status, and survival duration (Fig. 1E). Mining of a chromatin immunoprecipitation sequencing (ChIP-seq) dataset (GSE209112) revealed that FOXC1 was endogenously enriched on promoter regions of *ASNS* or *GPT2* (Fig. 1F). Dual-luciferase reporter, chromatin immunoprecipitation (ChIP), and Western blotting studies uncovered that steady overexpression or silencing of *FOXC1* into NB cells led to an increase or decrease in FOXC1 activity (Fig. S1E), FOXC1 enrichment (Fig. 1G), and protein levels of *ASNS* or *GPT2* (Fig. 1H and Fig. S1F). In cultured PC-3 and HeLa cells, ectopic expression or knockdown of *FOXC1* also resulted in the up-regulation or down-regulation of *ASNS* or *GPT2*, respectively (Fig. S1G and H). Of note, the levels of asparagine, alanine, and ^13^C_5_-glutamine metabolites were increased or reduced within SH-SY5Y and SK-N-BE(2) cell lines that were permanently transfected by *FOXC1* or short hairpin RNAs (shRNAs) targeting *FOXC1* (sh-FOXC1, Fig. 1I to K). There was an increase or decrease in asparagine and alanine levels in PC-3 and HeLa cells with overexpression or silencing of *FOXC1* (Fig. S1I). Interestingly, EBSS treatment facilitated FOXC1 protein levels in NB cells (Fig. S1J). The above findings suggested that *FOXC1* facilitated asparagine and alanine biogenesis in tumor cells.

### *FOXC1* promotes ferroptosis repression and NB progression via asparagine/alanine-facilitated CBS and FTH1 synthesis

**Fig. 2.**
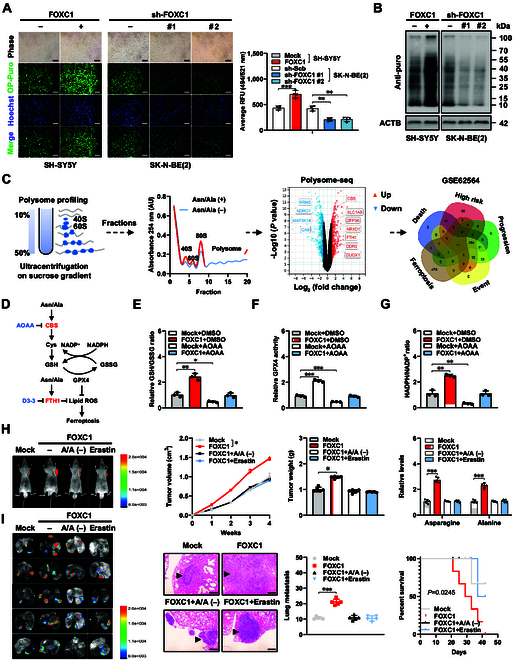
*FOXC1* promotes ferroptosis repression and NB progression via asparagine/alanine-facilitated CBS and FTH1 synthesis. (A and B) OP-puro (A) and puromycin (B) incorporation assays in SH-SY5Y or SK-N-BE(2) cells stably transfected with empty vector (mock), *FOXC1*, scramble shRNA (sh-Scb), sh-FOXC1 #1, or sh-FOXC1 #2. Scale bars: 10 μm. (C) Schematic illustration (left panel), sucrose gradient sedimentation (middle panel), volcano plot (middle panel), and Venn diagram (right panel) indicating the polysome profiling of mRNA translation altered in SK-N-BE(2) cells with asparagine/alanine (Asn/Ala) supplement or deprivation, and those associated with ferroptosis or death, high risk, clinical progression, and event in 498 NB cases (GSE62564). (D) Schematic illustration showing the impact of CBS, FTH1, and their inhibitors (AOAA or D3-3) on ferroptosis as indicated. (E to G) Relative GSH/GSSG ratio (E), GPX4 activity (F), and NADPH/NADP^+^ ratio (G) in SH-SY5Y cells stably transfected with mock or *FOXC1*, and those treated with DMSO or AOAA (1.0 μmol·l^−1^). (H) In vivo imaging (left panel), growth curve (middle panel), weight at the end points (middle panel), and asparagine and alanine levels (right panel) of xenograft tumors in nude mice formed by subcutaneous injection of SH-SY5Y cells stably transfected with mock or *FOXC1*, and those treated with asparagine/alanine (A/A)-free diet or erastin (0.5 nmol·l^−1^ per mouse, *n* = 5 for each group). (I) In vivo imaging (left panel), HE staining and quantification of lung metastasis (middle panels), and Kaplan–Meier curves (right panel) of nude mice treated with vein tail injection of SH-SY5Y cells stably transfected with mock or *FOXC1*, and those treated with A/A-free diet or erastin (0.5 nmol·l^−1^ per mouse, *n* = 5 for each group). Student’s *t* test or ANOVA compared the difference in (A) and (E) to (I). Log-rank test for survival comparison in (I). *P < 0.05, **P < 0.01, ***P < 0.001. Data are shown as mean ± SEM (error bars) or representative of 3 independent experiments in (A) to (I).

Since asparagine and alanine are essential amino acids for protein synthesis [29,30], O-propargyl-puromycin (OP-Puro) and puromycin incorporation assays were performed to reveal the increase or decrease of global protein synthesis within SH-SY5Y and SK-N-BE(2) cells exhibiting excessive expression or knockdown of *FOXC1*, respectively (Fig. 2A and B). In polysome profiling assay, asparagine and alanine supplement led to 618 increased and 566 decreased translating mRNAs associated with polysomes in lysates of SK-N-BE(2) cells (Fig. 2C and Tables S2 and S3), while 11 of them were substantially associated with ferroptosis and death, high risk, clinical progression, or events in 498 (GSE62564) cases of NB (Fig. 2C), including CBS and FTH1 (Fig. 2D). Consistent with the roles of CBS in facilitating GSH synthesis and GPX4 activity [11], stable ectopic expression of *FOXC1* increased the GSH/GSSG ratio (Fig. 2E), GPX4 activity (Fig. 2F), or NADPH/nicotinamide adenine dinucleotide phosphate (NADP^+^) ratio (Fig. 2G) in SH-SY5Y cells, which were abolished by treatment with aminooxyacetic acid (AOAA), an established inhibitor of CBS (Fig. 2E to G), without alteration in *CBS* or *FTH1* transcript levels (Fig. S1K). In addition, the decrease of free liable iron levels induced by *FOXC1* overexpression was reversed by treatment with FTH1 inhibitor D3-3 [31] (Fig. S1L).

Rescue studies were carried out to disclose the contribution of asparagine and alanine to *FOXC1*-induced tumor growth or aggressiveness. Asparagine and alanine deprivation abolished the elevation in GSH/GSSG ratio, GPX4 activity, or NADPH/NADP^+^ ratio, reduction in lipid ROS levels, and increase in mitochondrial membrane potential, proliferation, and invasion of NB cells exhibiting excessive expression of *FOXC1* (Fig. S2A to G). To evaluate the influence of *FOXC1* on the formation of tumors, SH-SY-5Y cells were injected either under the skin or into the tail vein of athymic nude mice. Small animal imaging test showed the increase of fluorescence levels in subcutaneous tumors produced by NB cells receiving stable *FOXC1* transfection (Fig. 2H). There was a notable increase in growth, mass, target gene (*ASNS*, *GPT2*, *CBS*, and *FTH1*) expression, Ki-67 expression, CD31-staining microvessels, and asparagine or alanine levels in xenograft tumors implanted under the skin of nude mice generated by SH-SY5Y cells with consistent transfection of *FOXC1* (Fig. 2H and Fig. S3A and B). In the experimental metastasis assay, athymic mice injected with SH-SY-5Y cells overexpressing *FOXC1* via the tail vein showed stronger fluorescence signals, more lung metastasis, and less survival rates (Fig. 2I). In the meantime, these in vivo changes caused by *FOXC1* overexpression were reversed by administration of asparagine- and alanine-free diet or erastin (Fig. 2H and I and Fig. S3A and B). These findings showed that *FOXC1* enhanced ferroptosis repression and NB progression via asparagine/alanine-facilitated CBS and FTH1 synthesis.

### OGT physically interacts with FOXC1 in NB cells

**Fig. 3.**
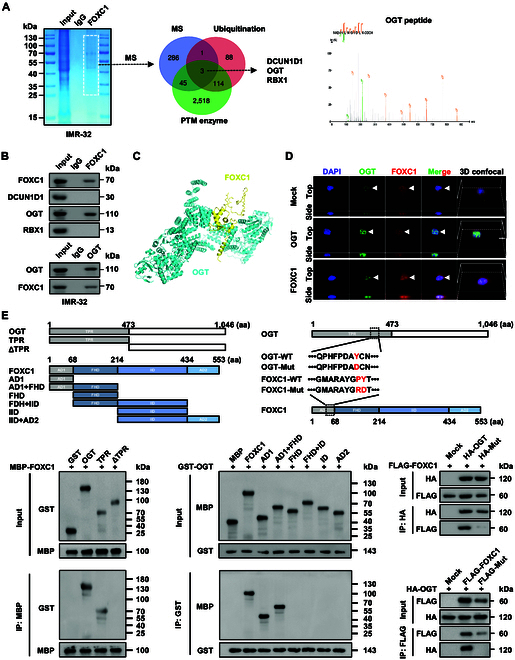
OGT physically interacts with FOXC1 in NB cells. (A) SDS-PAGE and Coomassie blue staining (left panel) of proteins immunoprecipitated by FOXC1 antibody from IMR-32 cell lysates, while Venn diagram (right panel) showing identification of FOXC1 protein partner by overlapping analysis of mass spectrometry (MS) results with those involved in deubiquitination process and PTM enzymes derived from the AmiGO2 (http://amigo.geneontology.org/) database. (B) Co-IP and Western blot assays indicating the interaction of FOXC1 with DCUN1D1, OGT, or RBX1 in IMR-32 cells. (C) Analysis of 3-dimensional (3D) structures of OGT and FOXC1 via the HDOCK program (http://hdock.phys.hust.edu.cn). (D) Immunofluorescent observation revealing colocalization of OGT and FOXC1 proteins (arrowheads) in SH-SY5Y cells transfected with empty vector (mock), *OGT*, or *FOXC1*. Scale bars: 10 μm. (E) Co-IP and Western blot assays (bottom panels) indicating the interaction between wild-type or truncations of recombinant GST-tagged *OGT* and MBP-tagged *FOXC1* proteins, and that in SH-SY5Y cells transfected with wild-type (WT) or mutant (Mut) HA-tagged *OGT* and FLAG-tagged *FOXC1* truncations as indicated (top panels). Data are shown as mean ± SEM (error bars) or representative of 3 independent experiments in (B), (D), and (E).

To determine the crucial FOXC1 protein partners, co-immunoprecipitation (co-IP) using FOXC1 antibody and subsequent proteomics tests were conducted, identifying 335 candidates within IMR-32 cell lysates (Fig. 3A and Table S4). Based on the evidence showing an increase in FOXC1 protein levels due to amino acid deprivation, we hypothesized that its protein partner involved in posttranslational modification (PTM) might regulate this process. By comparing mass spectrometry data with those essential for deubiquitination process and PTM enzymes from the AmiGO2 database (http://amigo.geneontology.org/), 3 proteins were identified, including defective in cullin neddylation 1 domain containing 1 (DCUN1D1), OGT, and ring box protein 1 (RBX1, Fig. 3A). Validating co-IP along with molecular docking studies indicated the binding of FOXC1 to OGT, but not to DCUN1D1 or RBX1, in IMR-32 cells (Fig. 3B and C). Immunofluorescence test indicated nuclear colocalization of OGT and FOXC1 in SH-SY-5Y cells, which was promoted by overexpression of either *OGT* or *FOXC1* (Fig. 3D). By using recombinant glutathione *S*-transferase (GST)-tagged OGT and maltose binding protein (MBP)-tagged FOXC1 proteins, co-IP as well as Western blotting studies showed that the tetratricopeptide repeat (TPR) domain (1 to 473 amino acids) of OGT and activation domain 1 (AD1, 1 to 68 amino acids) of FOXC1 were essential for their interaction (Fig. 3E), which was further validated in SH-SY5Y cells transfected by truncation constructs of hemagglutinin (HA)-tagged *OGT* and Flag-tagged *FOXC1* (Fig. S4A and B). In a bimolecular fluorescence complementation (BiFC) experiment, a physical interplay between OGT and FOXC1 was observed within the SH-SY5Y cell line transfected by their corresponding constructs (Fig. S4C). According to structural analysis using the HDOCK program [32], mutation of the 296th residue in the TPR domain of OGT or 66th and 67th residues in the AD1 domain of FOXC1 disrupted their interaction (Fig. 3E and Fig. S4C). These findings showed that there was a physical interaction between OGT and FOXC1 in NB cells.

### Liquid OGT/FOXC1 condensates promote asparagine/alanine-mediated ferroptosis repression and NB progression

**Fig. 4.**
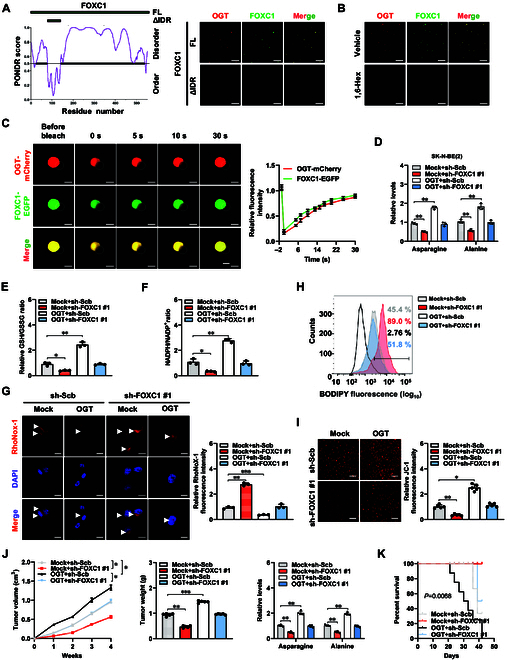
Liquid OGT/FOXC1 condensates promote asparagine/alanine-mediated ferroptosis repression and NB progression. (A) IDR within FOXC1 protein analyzed by the PONDR (http://www.pondr.com/) program (bottom left panel), with fluorescence imaging assay indicating the condensate formation of recombinant OGT-mCherry proteins and full-length (FL) or IDR-deficient (ΔIDR) FOXC1-EGFP (right panel) as indicated (top left panel). (B) Fluorescence imaging assay showing the condensate formation of recombinant OGT-mCherry and FOXC1-EGFP proteins, in the presence or absence of 1.5% 1,6-Hex. (C) Representative images (left panel) and quantification (right panel) of FRAP assay indicating the exchange kinetics of OGT-mCherry and FOXC1-EGFP within condensates. Scale bars: 10 μm. (D to F) Relative asparagine and alanine levels (D), GSH/GSSG ratio (E), and NADPH/NADP^+^ ratio (F) in SK-N-BE(2) cells stably transfected with empty vector (mock) or *OGT*, and those transfected with scramble shRNA (sh-Scb) or sh-FOXC1 #1 (*n* = 3). (G) Representative images (left panel) and quantification (right panel) of RhoNox-1 staining in SK-N-BE(2) cells stably transfected with mock or *OGT*, and those stably transfected with sh-Scb or sh-FOXC1 #1 (*n* = 3). Scale bars: 10 μm. (H) Flow cytometry showing the lipid ROS levels in SK-N-BE(2) cells stably transfected with mock or *OGT*, and those stably transfected with sh-Scb or sh-FOXC1 #1 (*n* = 3). (I) Representative images (left panel) and quantification (right panel) of JC-1 (2 μg·ml^−1^) staining in SK-N-BE(2) cells stably transfected with mock or *OGT*, and those stably transfected with sh-Scb or sh-FOXC1 #1 (*n* = 5). Scale bars: 10 μm. (J) Growth curve (left panel), weight at the end points (middle panel), and asparagine or alanine levels (right panel) of subcutaneous xenograft tumors in nude mice formed by SK-N-BE(2) cells stably transfected with mock or *OGT*, and those transfected with sh-Scb or sh-FOXC1 #1 (*n* = 5 for each group). (K) Kaplan–Meier curves of nude mice treated with tail vein injection of SK-N-BE(2) cells stably transfected with mock or *OGT*, and those transfected with sh-Scb or sh-FOXC1 #1 (*n* = 5 for each group). ANOVA compared the difference in (D) to (G), (I), and (J). Log-rank test for survival comparison in (K). *P < 0.05, **P < 0.01, ***P < 0.001. Data are shown as mean ± SEM (error bars) or representative of 3 independent experiments in (A) to (K).

Following the identification of intrinsically disordered regions (IDRs), consisting of 1 to 78 and 141 to 553 amino acid residues, within FOXC1 protein using the Preditor of Natural Disordered Regions program [33] (Fig. 4A), further studies on the potential liquid–liquid phase separation (LLPS) of FOXC1 and OGT proteins were carried out. In vitro droplet formation test of recombinant OGT (mCherry-tagged) and FOXC1 (mEGFP-tagged) proteins (purity>90%) showed their aggregation into droplets, resembling the compartment observed in the SK-N-AS cell line, which was abated following deletion of FOXC1 IDR or treatment with 1,6-hexanediol (1,6-Hex), an established inhibitor of LLPS [34] (Fig. 4A and B and Fig. S5A and B). The fluorescence recovery after photobleaching (FRAP) assay was used to investigate the fluid characteristics of OGT and FOXC1 within condensates, showing quick exchange kinetics (Fig. 4C and Fig. S5C). There was a decrease in FOXC1 activity, enrichment, and transcript or protein levels of *ASNS* and *GPT2* in SK-N-AS cells with stable *OGT* silencing, accompanied by down-regulation of CBS and FTH1, while ectopic expression of *FOXC1* prevented this effect (Fig. S6A to C). Meanwhile, the interplay of *OGT* and *FOXC1* in gene expression was abolished by 1,6-Hex treatment (Fig. S6A to C).

We next investigated functional interplay of *OGT* and *FOXC1* in asparagine/alanine biogenesis essential for ferroptosis repression and tumor progression. Stable ectopic expression of *OGT* elevated the asparagine and alanine levels (Fig. 4D), GSH/GSSG ratio (Fig. 4E), or NADPH/NADP^+^ ratio (Fig. 4F); reduced the free liable iron and lipid ROS levels (Fig. 4G and H); and increased the mitochondrial membrane potential in SK-N-BE(2) cells (Fig. 4I), whereas these effects were reversed by lowering *FOXC1* levels (Fig. 4D to I). The proliferative and invasive capabilities of SK-N-BE(2) cells were enhanced following forced excessive expression of *OGT*, but these effects were reversed when *FOXC1* was silenced (Fig. S6D and E). In nude mice, stable overexpression of *OGT* into SK-N-BE(2) cells facilitated the growth, weight, downstream gene (*ASNS*, *GPT2*, *CBS*, and *FTH1*) expression, Ki-67 expression, CD31-staining microvessels, and asparagine or alanine abundance of subcutaneous xenograft tumors, which were hindered due to silencing of *FOXC1* (Fig. 4J and Fig. S7A to C). Athymic nude mice injected with SK-N-BE(2) cells with excessive *OGT* expression via tail vein had increased lung metastatic colonies and a lower chance of survival. However, these effects were reversed when *FOXC1* was knocked down (Fig. 4K and Fig. S7D). These findings suggested that liquid FOXC1/OGT condensates promoted asparagine/alanine-mediated ferroptosis repression and NB progression.

### OGT stabilizes FOXC1 protein via O-GlcNAcylation in NB cells

**Fig. 5.**
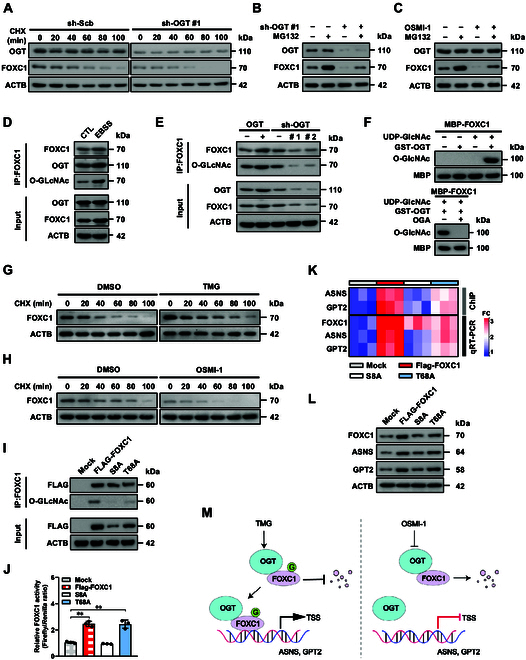
OGT stabilizes FOXC1 protein via O-GlcNAcylation in NB cells. (A) Western blot assay showing the FOXC1 levels in SH-SY5Y cells stably transfected with scramble shRNA (sh-Scb) or sh-OGT #1, and those treated with CHX (20 μg·ml^−1^) as indicated. (B and C) Western blot assay indicating the expression of OGT and FOXC1 in SH-SY5Y cells stably transfected with sh-Scb or sh-OGT #1 (B) or incubated with OSMI-1 (C, 2.0 μmol·l^−1^), and those treated with MG132 (10 μmol·l^−1^) for 4 h. (D) Co-IP and Western blot assays revealing the binding of OGT to FOXC1 and FOXC1 O-GlcNAcylation in SH-SY5Y cells treated with control (CTL) DMEM/F12 or EBSS for 6 h. (E) Co-IP and Western blot assays showing the O-GlcNAcylation of FOXC1 in SH-SY5Y cells stably transfected with empty vector (mock), OGT, sh-Scb, sh-OGT #1, or sh-OGT #2. (F) In vitro O-glcNAcylation assay indicating the O-glcNAcylation of MBP-tagged FOXC1 protein in the presence of UDP-GlcNAc, GST-tagged OGT, or OGA. (G and H) Western blot assay showing the FOXC1 levels in SK-N-BE(2) (G) and SH-SY5Y (H) cells treated with TMG (G, 10 μmol·l^−1^) or OSMI-1 (H, 2.0 μmol·l^−1^), and those incubated with CHX (20 μg·ml^−1^) as indicated. (I) Co-IP and Western blot assays revealing the O-GlcNAcylation of FOXC1 in SH-SY5Y cells stably transfected with mock, Flag-tagged *FOXC1*, and that containing S8A or T68A mutation. (J to L) Dual-luciferase (J), ChIP-qPCR (K, normalized to input, *n* = 3), real-time qRT-PCR (K, normalized to *β-actin*, *n* = 5), and Western blot (L) assays showing the FOXC1 activity, FOXC1 enrichment, and target gene (*ASNS* or *GPT2*) expression in SH-SY5Y cells stably transfected with mock, Flag-tagged *FOXC1*, and that containing S8A or T68A mutation. (M) Schematic illustration indicating the action modes of OGT-mediated O-GlcNAcylation (G) of FOXC1 in regulating target gene (*ASNS* or *GPT2*) expression. ANOVA compared the difference in (J). **P < 0.01. Data are shown as representative of 3 independent experiments in (A) to (L).

To investigate the mechanisms regulating FOXC1 expression, we further observed the impact of OGT on FOXC1 protein stability. Stable silencing of *OGT* resulted in the decrease of FOXC1 levels within the SH-SY5Y cell line pretreated by cycloheximide (CHX), an acknowledged protein synthesis inhibitor (Fig. 5A). In addition, a proteasome inhibitor (MG132) was able to rescue the decrease of FOXC1 protein levels in NB cells treated with *OGT* knockdown or OGT inhibitor (OSMI-1) [35] (Fig. 5B and C). Interestingly, EBSS treatment substantially increased the OGT binding and O-GlcNAcylation of FOXC1 in the SK-N-BE(2) cell line (Fig. 5D). Increased or reduced O-GlcNAcylation and expression levels of FOXC1 were observed within NB cell lines with excessive expression or silencing of *OGT* (Fig. 5E). To further assess O-GlcNAcylation of FOXC1 protein, in vitro O-GlcNAcylation assay was performed, which revealed the direct O-GlcNAcylation of MBP-tagged FOXC1 protein by GST-tagged OGT, while incubation of O-GlcNAcase (OGA) abolished these effects (Fig. 5F). Notably, the stability of FOXC1 protein was facilitated or decreased in SK-N-BE(2) and SH-SY5Y lines receiving treatment of TMG (an activator of O-GlcNAcylation) [36] or OSMI-1, respectively (Fig. 5G and H). Next, we further explored the amino acid residues essential for O-GlcNAcylation of FOXC1. Based on analysis using the O-GlcNAcPRED-DL tool [37], mutation of the 8th serine, but not of the 68th threonine, of FOXC1 led to a decrease of its O-GlcNAcylation, transcriptional activity, and enrichment on target genes, resulting in the down-regulation of *ASNS* and *GPT2* in NB cells (Fig. 5I to L). These results suggested that OGT stabilized FOXC1 protein via O-GlcNAcylation (Fig. 5M) in NB cells.

### *EcircOGT*-encoded OGT-570aa inhibits asparagine/alanine biogenesis and ferroptosis repression in NB cells

**Fig. 6.**
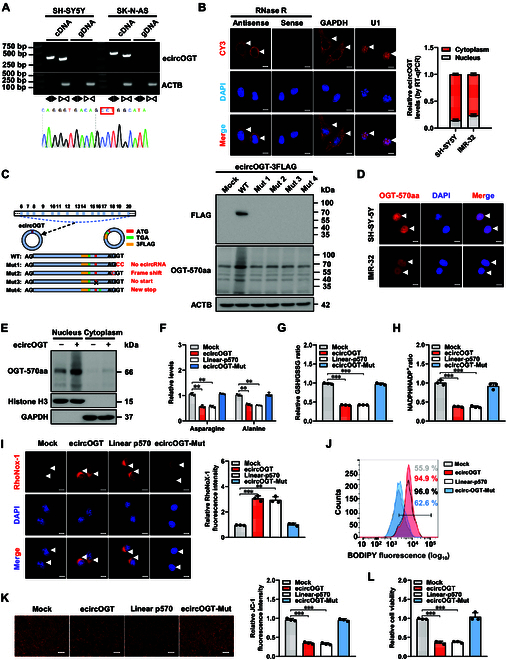
*EcircOGT*-encoded OGT-570aa inhibits asparagine/alanine biogenesis and ferroptosis repression of NB cells. (A) Validation of *ecircOGT* in cDNA or genomic DNA (gDNA) using convergent or divergent primers (top panel) and Sanger sequencing (bottom panel). (B) RNA-FISH (left panel) assay showing cytoplasmic and nuclear localization of *ecircOGT* in cultured SH-SY5Y cells using a junction-specific antisense probe (red), with nuclei staining with DAPI (blue). Sense probe with RNase R (3 U·mg^−1^) treatment was used as a negative control. The *GAPDH* and *U1* were applied as positive controls. Scale bar: 10 μm. Real-time qRT-PCR (right panel, normalized to *β-actin*) assay indicating the distribution of *ecircOGT* in cytoplasmic and nuclear fractions of SH-SY5Y cells (*n* = 4). (C) Schematic illustration (left panel) and Western blot (right panel) assay showing the expression of *ecircOGT*-encoded protein (OGT-570aa) in IMR-32 cells transfected with empty vector (mock), 3×Flag-tagged *ecircOGT*, or 3×Flag-tagged *ecircOGT* with circularization site or ORF mutation (Mut). (D) Immunofluorescence assay revealing nuclear colocalization of OGT-570aa in SH-SY5Y cells, with nuclei stained by DAPI (blue). Scale bar: 10 μm. (E) Western blot assay revealing the levels of OGT-570aa in cytoplasmic and nuclear fractions of IMR-32 cells stably transfected with mock or 3×Flag-tagged *ecircOGT*. (F to H) Relative asparagine and alanine levels (F), GSH/GSSG ratio (G), and NADPH/NADP^+^ ratio (H) in IMR-32 cells stably transfected with mock, *ecircOGT*, linear p570 construct, or *ecircOGT* with circularization site mutation (*ecircOGT* Mut, *n* = 3). (I) Representative images (left panel) and quantification (right panel) of RhoNox-1 staining in IMR-32 cells stably transfected with mock, *ecircOGT*, linear p570 construct, or *ecircOGT* Mut (*n* = 3). Scale bars: 10 μm. (J) Flow cytometry showing the lipid ROS levels in IMR-32 cells stably transfected with mock, *ecircOGT*, linear p570 construct, or *ecircOGT* Mut (*n* = 3). (K) Representative images (left panel) and quantification (right panel) of JC-1 (2 μg·ml^−1^) staining in IMR-32 cells stably transfected with mock, *ecircOGT*, linear p570 construct, or *ecircOGT* Mut (*n* = 3). (L) MTT colorimetric assay indicating the viabilities of IMR-32 cells stably transfected with mock, *ecircOGT*, linear p570 construct, or *ecircOGT* Mut (*n* = 3). ANOVA compared the difference in (F) to (I), (K) and (J). **P < 0.01, ***P < 0.001. Data are shown as mean ± SEM (error bars) or representative of 3 independent experiments in (A) to (L).

To identify the endogenous regulator of OGT activity, considering the roles of circular RNA (circRNA)-generated proteins in regulating the functions of its host gene [38], we performed the mining of circBase [39] and open reading frame (ORF) finder (https://www.ncbi.nlm.nih.gov/orffinder) databases, which revealed an *OGT*-derived circRNA (*hsa_circ_0091037*, referred to as *ecircOGT*) containing an ORF spanning junction. The precise circularization of *ecircOGT* originating from exons 6 to 20 of *OGT* was confirmed in SH-SY5Y and SK-N-AS cell lines (Fig. 6A), showing traits of cytoplasmic abundance and RNase R resistance (Fig. 6B). *EcircOGT* was less abundant in NB cell lines or clinical specimens of different International Neuroblastoma Staging System (INSS) stages, in comparison to normal dorsal root ganglia (Fig. S7E and F). Overexpression of *ecircOGT* did not impact the *OGT* expression in NB cells (Fig. S7G). Meanwhile, eukaryotic translation initiation factor 4A3 (EIF4A3), a crucial controller of circRNA biogenesis [40], was elevated in NB cell lines and tissues (Fig. S7F). In line with data from the ENCORI database (https://rnasysu.com/encori/), RNA immunoprecipitation (RIP) test showed EIF4A3 enrichment near exons 6 and 20 of *OGT*, which was reduced by *EIF4A3* knockdown (Fig. S7H). Of note, suppressing *EIF4A3* expression led to higher *ecircOGT* levels in NB cells, without affecting *OGT* expression (Fig. S7I). The expression of a protein with 570 amino acid residues (OGT-570aa), containing a partial TPR domain (382 to 473 aa) of OGT along with 3 extra residues (CHP), was detected in the IMR-32 cell line receiving transfection with FLAG-tagged *ecircOGT*, but was not found in that with mutation of circularization site or ORF (Fig. 6C). The protein was mainly located in the nucleus (Fig. 6D and E). The asparagine and alanine levels, GSH/GSSG ratio, and NADPH/NADP^+^ ratio were decreased, while free liable iron and lipid ROS levels were increased, and the mitochondrial membrane potential and viabilities of IMR-32 cell line were reduced by transfection of *ecircOGT* or its linear ORF, but not by that containing mutation of circularization site (*circOGT1* Mut, Fig. 6F to L). These results indicated that *ecircOGT*-encoded OGT-570aa repressed asparagine/alanine biogenesis and ferroptosis repression in NB cells.

### OGT-570aa inhibits ferroptosis repression and NB progression via blocking OGT–FOXC1 interaction

**Fig. 7.**
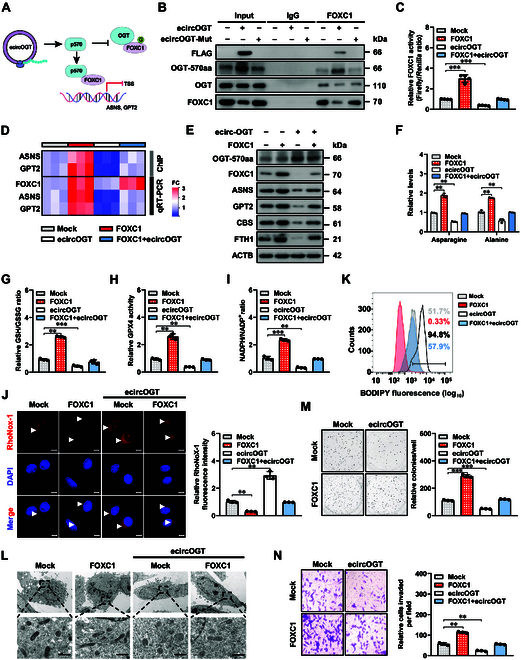
OGT-570aa inhibits ferroptosis repression and NB progression via blocking OGT–FOXC1 interaction. (A) Schematic illustration indicating the action modes of *ecircOGT*-encoded p570 in regulating OGT–FOXC1 interaction and FOXC1 target gene (*ASNS* or *GPT2*) expression. (B) Co-IP and Western blot assays showing the interaction among OGT-570aa, OGT, and FOXC1 in IMR-32 cells stably transfected with empty vector (mock), *ecircOGT*, or *ecircOGT* Mut. (C to E) Dual-luciferase (C), ChIP and qPCR (D, normalized to input), real-time qRT-PCR (D, normalized to *β-actin*), and Western blot (E) assays showing the FOXC1 activity, FOXC1 enrichment, and downstream gene (*ASNS*, *GPT2*, *CBS*, and *FTH1*) expression in IMR-32 cells stably transfected with mock, *FOXC1*, or *ecircOGT*. (F to I) Relative asparagine and alanine levels (F), GSH/GSSG ratio (G), GPX4 activity (H), and NADPH/NADP^+^ ratio (I) in IMR-32 cells stably transfected with mock, *FOXC1*, or *ecircOGT* (*n* = 3). (J) Representative images (left panel) and quantification (right panel) of RhoNox-1 staining in IMR-32 cells stably transfected with mock, *FOXC1*, or *ecircOGT* (*n* = 3). Scale bars: 10 μm. (K) Flow cytometry showing the lipid ROS levels in IMR-32 cells stably transfected with mock, *FOXC1*, or *ecircOGT* (*n* = 3). (L) Representative images of mitochondria shrunk in IMR-32 cells stably transfected with mock, *FOXC1*, or *ecircOGT.* Scale bars: 0.5 μm. (M) and (N) Representative images (left panel) and quantification (right panel) of soft agar (M) and matrigel invasion (N) in IMR-32 cells stably transfected with mock, *FOXC1*, or *ecircOGT* (*n* = 3). ANOVA compared the difference in (C), (F) to (I), (J), (M), and (N). **P < 0.01, ***P < 0.001. Data are shown as mean ± SEM (error bars) or representative of 3 independent experiments in (B) to (N).

Since the above evidence revealed that OGT-570aa contained partial TPR domain essential for binding of OGT to FOXC1, we further investigated the hypothesis that it might influence the OGT–FOXC1 interaction (Fig. 7A). Ectopic expression of *ecircOGT*, but not of *ecircOGT* Mut, increased the binding of OGT-570aa with FOXC1, leading to reduced OGT–FOXC1 interaction in IMR-32 cells (Fig. 7B). Of note, stable overexpression of *ecircOGT* decreased FOXC1 activity (Fig. 7C) and reduced FOXC1 enrichment and transcript or protein levels of *ASNS* and *GPT2* in IMR-32 cells, along with a decrease in CBS and FTH1 expression (Fig. 7D and E), which were rescued by ectopic expression of *FOXC1* (Fig. 7C to E). In addition, transfection of *FOXC1* prevented the reduction in asparagine and alanine levels, GSH/GSSG ratio, GPX4 activity, or NADPH/NADP^+^ ratio; an increase in free liable iron, lipid ROS levels, and mitochondria; and a decrease in growth and invasiveness of IMR-32 cells exhibiting stable *ecircOGT* overexpression (Fig. 7F to N and Fig. S7J). Together, these results showed that OGT-570aa repressed ferroptosis repression and aggressiveness of NB cells via blocking OGT–FOXC1 interaction.

### MN suppresses NB progression via facilitating OGT-570aa–FOXC1 interaction

**Fig. 8.**
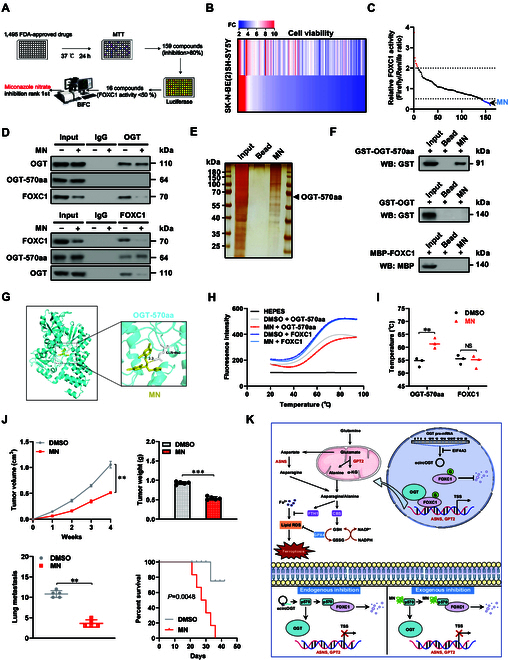
Miconazole nitrate (MN) suppresses NB progression via facilitating OGT-570aa–FOXC1 interaction. (A) Schematic illustration indicating the screening of FOXC1 inhibitor from 1,495 FDA-approved drugs by MTT colorimetric, dual-luciferase, and BiFC assays. (B) MTT colorimetric assay showing the viabilities of SH-SY5Y and SK-N-BE(2) cells treated with 159 compounds. (C) Dual-luciferase assay revealing the FOXC1 activity in SK-N-BE(2) cells treated with 1,495 FDA-approved drugs, with 16 of them possessing inhibitory ratios larger than 50%. (D) Co-IP and Western blot assays showing the interaction among OGT-570aa, OGT, and FOXC1 in SK-N-BE(2) cells treated with solvent or MN. (E and F) Silver staining (E) and Western blot (F) assays indicating recombinant proteins with affinity to FG beads covalently conjugated with MN (20 μmol·l^−1^). (G) Molecular docking analysis of OGT-570aa and MN via the HDOCK program (http://hdock.phys.hust.edu.cn/). (H and I) DSF assay (H) and temperature (I) showing the fluorescence intensity in HEPES, solvent (DMSO), MN, OGT-570aa, or FOXC1. (J) Growth curve, weight at the end points of xenograft tumors, lung metastatic counts, and Kaplan–Meier curves of nude mice treated with subcutaneous or tail vein injection of SK-N-BE(2) cells, and treated with DMSO or MN (50 mg·kg^−1^, *n* = 5 for each group). (K) Schematic depicting the mechanisms underlying OGT-570aa/*OGT*/*FOXC1*-regulated tumor progression: as a transcription factor, FOXC1 facilitates the expression of asparagine/alanine biogenesis genes (*ASNS* and *GPT2*), while OGT interacts with FOXC1 to facilitate its O-GlcNAcylation and stability, resulting in increase of asparagine or alanine, and up-regulation of CBS and FTH1 essential for ferroptosis resistance. Meanwhile, as an EIF4A3-repressed circRNA derived from *OGT*, *ecircOGT* is able to encode a novel protein (OGT-570aa) containing a partial TPR domain of OGT protein, which competitively binds with FOXC1 and blocks OGT–FOXC1 interaction. Administration of MN facilitates the binding of OGT-570aa to FOXC1, resulting in repression of *ASNS* or *GPT2* expression, ferroptosis resistance, tumorigenesis, and aggressiveness. Student’s *t* test or ANOVA compared the difference in (I) and (J). Log-rank test for survival comparison in (J). **P < 0.01, ***P < 0.001. NS, nonsignificant. Data are shown as mean ± SEM (error bars) or representative of 3 independent experiments in (D) to (J).

To screen chemical inhibitor of FOXC1 activity, NB cells were treated with 1,495 Food and Drug Administration (FDA)-approved drugs (Fig. 8A), while 159 compounds with inhibition ratios of more than 80% on cellular viabilities were obtained (Fig. 8B), which were subjected to dual-luciferase assay for observing their effects on FOXC1 activity. The results indicated that 17 chemicals were able to suppress FOXC1 activity with efficiency larger than 80% (Fig. 8C). Among them, canagliflozin, dasatinib, indacaterol, menadione, MN, paroxetine HCl, proguanil, trifluoperazine, tegaserod maleate, and vortioxetine suppressed the OGT–FOXC1 interaction in BiFC assay, with MN ranking first in inhibitory effects (Fig. S8A). Notably, the viabilities of HEK293 cells were not impacted by MN (Fig. S8B). In co-IP and Western blot experiments, MN substantially facilitated the binding of OGT-570aa to FOXC1, resulting in a decrease of OGT–FOXC1 interaction and the down-regulation of FOXC1 (Fig. 8D). The use of ferrite-glycidyl methacrylate (FG) beads [41] in affinity purification showed a specific interaction of MN with recombinant OGT-570aa, but not with OGT or FOXC1 protein (Fig. 8E and F). Molecular docking via the HDOCK program (http://hdock.phys.hust.edu.cn/) revealed direct interaction (distance, 3.3 Å) of MN with the 468th to 470th residues (GLY) of OGT-570aa (Fig. 8G), which was further validated by differential scanning fluorimetry (DSF) assay (Fig. 8H and I). The activity and enrichment of FOXC1 were decreased in NB cells treated with MN, leading to lower transcript and protein levels of *ASNS* and *GPT2*. This was accompanied by down-regulation of CBS and FTH1, but ectopic expression of *FOXC1* prevented these effects (Fig. S9A to C). Administration of MN reduced asparagine and alanine levels (Fig. S9D), GSH/GSSG ratio (Fig. S9E), or NADPH/NADP^+^ ratio (Fig. S9F); increased the free liable iron and lipid ROS levels (Fig. S9G and H); and attenuated the mitochondrial membrane potential (Fig. S9I), while excessive *FOXC1* expression reversed these effects (Fig. S9D to I). The proliferative and invasive capabilities of NB cell lines were hindered by treatment with MN, but this effect was reversed by the ectopic expression of *FOXC1* (Fig. S9J and K). In nude mice, administration of MN decreased the growth, mass, target gene expression, Ki-67 or CD31 levels, and asparagine and alanine levels of hypodermic xenograft tumors (Fig. 8J and Fig. S10A to D). Athymic nude mice receiving MN injection via the tail vein showed a lower number of lung metastatic colonies and increased survival rate in the experimental metastasis assay (Fig. 8J and Fig. S10E).

Higher levels of *OGT*, *FOXC1*, *ASNS*, *GPT2*, *CBS*, and *FTH1*, as well as lower expression of *ecircOGT* or OGT-570aa, were noted in NB samples from patients with advanced INSS stages (Fig. S11A and B). In Kaplan–Meier survival assay, elevated *OGT* (*P* = 2.7 × 10^−2^ and *P* = 1.0 × 10^−3^), *FOXC1* (*P* = 2.2 × 10^−14^ and *P* = 4.0 × 10^−2^), *ASNS* (*P* = 2.7 × 10^−7^ and *P* = 8.3 × 10^−3^), *GPT2* (*P* = 1.4 × 10^−15^ and *P* = 1.6 × 10^−9^), *CBS* (*P* = 1.5 × 10^−16^ and *P* = 1.1 × 10^−5^), and *FTH1* (*P* = 2.7 × 10^−4^ and *P* = 1.5 × 10^−2^) were associated with poorer prognosis in 498 (GSE62564) and 283 (GSE85047) NB cases (Fig. S11C). In 498 (GSE62564) NB specimens, the *ASNS* (*R* = 0.124, *P* = 5.5 × 10^−3^) or *GPT2* (*R* = 0.189, *P* = 2.1 × 10^−5^) levels were substantially correlated with those of *FOXC1* (Fig. S11D). In addition, higher expression of *OGT*, *FOXC1*, *ASNS*, *GPT2*, *CBS*, or *FTH1* was also associated with poorer outcomes in clinical cases with renal clear cell carcinoma or glioma (Fig. S12). These findings suggested that MN inhibited tumor progression by promoting the interaction between OGT-570aa and FOXC1.

## Discussion

Asparagine, a nonessential amino acid, is de novo synthesized through the transfer of an acylamino group from glutamine to aspirate, a process mediated by ASNS [29], and is required for protein or nucleotide synthesis [29]. Increased *ASNS* expression is linked to unfavorable outcome in multiple solid malignancies, including prostate cancer [42], HCC [43], and glioma [44]. Ectopic expression of *ASNS* rescues the apoptosis and cell cycle arrest induced by activating transcription factor 4 deficiency or nutrition stress [45]. Meanwhile, knockdown of *ASNS* reduces *MYC* expression and tumor growth [46], implicating *ASNS* as a possible focus for cancer treatment. GPT2 is a mitochondrial aminotransferase catalyzing the reversible reaction between pyruvate and glutamate to produce alanine and α-ketoglutarate [30]. In breast cancer, *GPT2* serves as a pivot between glycolysis and glutaminolysis, and promotes tumorigenesis and stemness [47]. Meanwhile, silencing of *GPT2* is able to suppress the proliferation of signet ring cell carcinoma [48] or pancreatic cancer [49], corroborating GPT2 as a critical enzyme in oncogenesis. In this research, we discovered that FOXC1 drove the transcription of genes (*ASNS* and *GPT2*) crucial for amino acid metabolism, and facilitated the production of asparagine and alanine, resulting in increase of CBS and FTH1 protein synthesis, ferroptosis resistance, tumorigenesis, and aggressiveness of NB. Furthermore, OGT directly interacted with FOXC1 to block its proteasomal degradation via mediating O-GlcNAcylation. Meanwhile, a novel OGT-570aa isoform encoded by *ecircOGT* was able to inhibit the interaction of OGT and FOXC1, thus promoting ferroptosis and decreasing tumorigenesis and aggressiveness (Fig. 8K), which suggests that targeting the *ecircOGT*/*OGT*/*FOXC1* axis could be an option for NB treatment.

OGT is a nutrient- or stress-responsive glycosyltransferase catalyzing the incorporation of N-acetylglucosamine (GlcNAc) from UDP-GlcNAc to serine or threonine residues [50], and regulates the structure, stability, or function of nuclear and cytoplasmic proteins, such as kinases, phosphatases, transcription factors, and metabolic enzymes [50]. Recent studies reveal that OGT-mediated O-GlcNAcylation regulates multiple phenotypes of tumorigenesis [51–54]. For example, the nuclear localization of serine/arginine-rich protein kinase 2 (SRPK2) is influenced by O-GlcNAcylation, promoting both de novo lipogenesis and tumorigenesis [51]. Glucose-triggered O-GlcNAcylation of phosphoribosyl pyrophosphate synthetase 1 (PRPS1) boosts generation of nucleotide and nicotinamide adenine dinucleotide (NAD) crucial for tumor growth [52]. O-GlcNAcylation of yes-associated protein (YAP) represses its interaction with large tumor suppressor gene 1 (LATS1), resulting in its transactivation and tumor progression [53]. In addition, O-GlcNAcylation stabilizes Sirtuin 7 by inhibiting its interaction with proteasome activator subunit 3, and promotes pancreatic cancer progression [54]. In this study, we found that OGT interacted with FOXC1 to mediate its O-GlcNAcylation, leading to its up-regulation in driving transcription of *ASNS* and *GPT2* in NB cells. Structurally, FOXC1 protein contains an N-terminal activation domain (AD) [16]. Our data indicated that TPR domain of OGT was able to interact with AD1 of FOXC1, while their underlying structures warrant further investigation.

CircRNAs are loops of single-stranded noncoding RNAs formed through back splicing [38]. EIF4A3, the core element of exon junction complex, is able to facilitate or repress the biogenesis of circRNAs [55,56]. For example, EIF4A3 promotes the circularization of *circIKBKB* [55], while it inhibits the expression of *circ_0087429* by binding to flanking regions [56]. In this study, we discovered that *ecircOGT* was a circRNA repressed by EIF4A3, but it did not alter the levels of parental gene *OGT* in NB. Recent studies show that circRNAs are crucial in the development and progression of cancer through sponging microRNAs or binding with RNA-binding proteins [38]. Some circRNAs like *circ-ZNF609*, *circFBXW7*, *circMbl*, and *circ-SHPRH* have the ability to produce peptides or proteins through internal ribosome entry site or N6-methyladenosine (m^6^A) modification [38]. For example, m^6^A modification facilitates the production of circMAP3K4-455aa in HCC, which protects cancer cells from cisplatin treatment and predicts worse prognosis of patients [57]. *CircCAPG* produces a new protein CAPG-171aa that boosts the growth and spread of triple-negative breast carcinoma [58]. *CircPPP1R12A* produces a protein that enhances colon cancer growth and spread by activation of the Hippo-YAP signaling pathway [59]. Meanwhile, *circZKSCAN1*-encoded peptide serves as a tumor suppressor and sensitizes HCC cells to sorafenib via ubiquitination of mammalian target of rapamycin [60]. In the current study, we found that *ecircOGT*-encoded OGT-570aa contained a partial TPR domain of OGT protein, which was efficient in promoting ferroptosis and decreasing tumor progression by competitively binding with FOXC1 and reducing interaction of OGT with FOXC1, indicating its importance as a therapeutic focus regulating ferroptosis and tumor progression.

MN is a broad-spectrum imidazole antifungal agent [61] and is able to suppress neuroinflammation [62]. Preliminary evidences indicate that MN also exerts anticancer effects in various solid tumors [63–66]. MN induces cell cycle arrest [63] and triggers apoptosis of bladder or colon cancer via the death receptor 5-dependent or mitochondrial-mediated pathway [63,64]. MN suppresses the production of vascular endothelial growth factor in gliomas or breast cancer by blocking the translation of hypoxia inducible factor 1 alpha [65]. Moreover, MN reduces global protein production by causing phosphorylation of translation initiation factor 2 alpha [65]. In osteosarcoma cells, MN represses the proliferation of tumor cells by increasing Ca^2+^ levels [66]. However, the anticancer mechanisms of MN are not well understood. In this research, we found that MN could facilitate the binding of OGT-570aa to FOXC1, thus reducing the biogenesis of alanine and asparagine, inducing ferroptosis, and inhibiting tumorigenesis and aggressiveness, which indicates its potential application as an inhibitor of tumor progression.

In summary, we show that enhanced FOXC1 levels are linked to a poor outcome for NB patients. FOXC1 is stabilized by OGT-mediated O-GlcNAcylation in liquid condensates to serve as a transcriptional activator of *ASNS* and *GPT2*, thereby increasing intracellular asparagine and alanine biogenesis, which enhances the ability of NB cells to resist ferroptosis, as well as promotes their growth, invasion, and metastasis. Meanwhile, *ecircOGT*, an EIF4A3-inhibited circRNA originating from *OGT*, can produce a new protein termed as OGT-570aa, which inhibits the interaction of OGT with FOXC1. Administration of MN facilitates the interaction of OGT-570aa with FOXC1, thus suppressing tumorigenesis and aggressiveness. We believe that this work reveals the crucial roles and mechanisms of *ecircOGT*/*OGT*/*FOXC1* axis in regulating ferroptosis, and provides new therapeutic strategies for tumors. More efforts are needed to reveal the additional functions and target genes of *FOXC1* during the progression of NB and other tumors. The prognostic values of the *ecircOGT*/*OGT*/*FOXC1* axis and downstream genes for NB warrant further investigation via using a larger series of cases with longer follow-up duration.

## Materials and Methods

### Culture of cell lines

The cell lines of NB [SK-N-DZ (CRL-2149), IMR-32 (CCL-127), SK-N-BE(2) (CRL-2271), SK-N-AS (CRL-2137), SH-SY5Y (CRL-2266), and SK-N-SH (HTB-11)], prostate cancer (PC-3, CRL-1435), cervical cancer (HeLa, CCL-2), and embryonic kidney (HEK293, CRL-1573) were acquired from the American Type Culture Collection (Rockville, MD) or the Pediatric Oncology Group Cell Bank (Lubbock, TX). Short tandem repeat analysis was used for verification of cell lines, which were used within 6 months. Regularly, the Mycoplasma PCR Detection Kit (ab289834, Abcam Inc., Cambridge, MA) was used to monitor mycoplasma contamination. The SK-N-DZ, IMR-32, SK-N-AS, SK-N-SH, HeLa, and HEK293 cells were cultured with Dulbecco’s Modified Eagle’s Medium (DMEM) (Invitrogen, Carlsbad, CA), while SK-N-BE(2) and SH-SY5Y cells were respectively grown in Minimum Essential Medium (MEM)/F12 or DMEM/F12 (Invitrogen) along with 10% fetal bovine serum (FBS, Sigma, St. Louis, MO). The PC-3 cells were grown in RPMI-1640 medium (Invitrogen) containing 10% FBS. Cells were exposed to EBSS (Sigma), erastin (MedChemExpress, Monmouth Junction, NJ), rapamycin (MedChemExpress), cisplatin (MedChemExpress), elesclomol (MedChemExpress), AOAA (MedChemExpress), D3-3 (MedChemExpress), 1,6-Hex (Sigma), MG132 (Sigma), Thiamet-G (TMG, Sigma), OSMI-1 (Sigma), or MN (MedChemExpress) at 37 °C in 5% CO_2_. Amino acid deprivation or supplement was achieved by formulating medium, according to manuals of manufacturers (Life Technologies Inc.).

### Real-time quantitative RT-PCR

RNAs from the entire cell, cytoplasm, or nucleus were obtained through the use of the PureLink RNA Mini Kit (Thermo Fisher Scientific Inc., Waltham, MA) or Cytoplasmic and Nuclear RNA Purification Kit (Norgen Biotek, Ontario, Canada). To detect circRNA, samples were digested by RNase R (3 U·μg^−1^, Abcam Inc.) for 15 min at 37 °C, followed by cDNA synthesis using a Reverse Transcription kit (QIAGEN, Germantown, MD). The SYBR Green Master Mix from Bio-Rad (Hercules, CA) and primers (Table S5) were utilized for mRNA and circRNA quantification [67–70].

### RNA fluorescence in situ hybridization

Tsingke Biotech Co., Ltd (Beijing, China) synthesized biotin-labeled antisense or sense probes (Table S6) targeting *ecircOGT* junction. The probes for glyceraldehyde 3-phosphate dehydrogenase (*GAPDH*) or U1 small nuclear 1 (*U1*) were created via the HighFidelity Digoxigenin PCR Labeling Kit from Jena Bioscience (Jena, Germany) and PCR products (Table S5). Cells were treated with probe (40 nmol·l^−1^) and incubated at 37 °C for 16 h, with or without RNase R (3 U·μg^−1^) treatment. The Ribo fluorescence in situ hybridization (FISH) Kit (RiboBio, Guangzhou, China) was employed to detect *ecircOGT*, while nuclei were stained by 4′,6-diamidino-2-phenylindole (DAPI) from Sigma [68,69].

### Western blot

Proteins were isolated by cell lysis buffer from Cell Signaling Technology Inc. (Danvers, MA). The Nuclear/Cytosol Fractionation kit (ab289882) was employed for segregating cytoplasmic or nuclear parts. Western blot procedure was carried out as previously explained [67–70], using antibodies designed specifically for FOXC1 (ab226219), OGT (ab177941), ASNS (ab126254), GPT2 (ab232963), CBS (ab96252), FTH1 (ab75972), DCUN1D1 (ab181233), RBX1 (ab133565), Myc-tag (ab9106), GST-tag (ab111947), β-actin (ab6276, Abcam Inc.), GAPDH (2118), histone H3 (9715), MBP-tag (2396), Flag-tag (2368), His-tag (2365), or HA-tag (2367, Cell Signaling Technology Inc., Danvers, MA). A polyclonal antibody of OGT-570aa was created by vaccinating rabbits with a synthetic peptide matching the C-terminal end of OGT-570aa (LWAGTPMVTMPGCHP) at ABclonal Biotechnology Co., Ltd in Wuhan, China.

### Ectopic expression or silencing of genes

Genechem Co., Ltd (Shanghai, China) supplied human *FOXC1* cDNA (1,662 bp) and *OGT* cDNA (3,131 bp), which were then inserted into CV186. The PCR primers (Table S6) were used to amplify their truncations, which were then inserted into various plasmids including pCMV-3Tag-1A, pMAL-c4X, pCMV-HA, pGEX-6P-1, pET28a-EGFP, pET28a-mCherry, and pmCherry-N1 from Addgene and Genprice Inc. The linear form of *ecircOGT* (*hsa_circ_0091037*) was amplified from NB specimens using specific primer sets (Table S6) and then inserted into pLCDH-ciR (Geneseed Biotech Co., Guangzhou, China). A construction of *ecircOGT*-3Flag was made by adding a 3×Flag-coding gene before the stop codon in linear *ecircOGT* (Table S6). The OGT-570aa-3Flag sequence (Table S6) was inserted into pcDNA3.1-mini or pGEX-6P-1 (Addgene) through subcloning. The QuikChange II Site-Directed Mutagenesis Kit (Agilent, Santa Clara, CA) was used along with primers (Table S6) to induce mutations in *FOXC1*, *OGT*, or ecircOGT constructs. Specific oligonucleotides targeting shRNAs (Table S7) were inserted into GV298 (Genechem Co., Ltd).

### Lentiviral packaging

The HEK293T cells were co-transfected by psPAX2 and pMD2G (Addgene) along with lentiviral vectors. Infectious lentivirus was extracted at 36 and 60 h posttransfection, filtered through 0.45-μm polyvinylidene difluoride filters (Sigma), and then diluted in phosphate-buffered saline (PBS).

### Dual luciferase reporter assay

A luciferase reporter of human FOXC1 activity was created by inserting oligonucleotides with 4 standard FOXC1 motifs (Table S6) into pGL4.1 vector (Promega, Madison, WI). The dual-luciferase assay was conducted using a GloMax Luminometer (Promega) [67–70].

### ChIP analysis

ChIP experiment was undertaken with the EZ-ChIP kit from MerckMillipore (Darmstadt, Germany) and antibodies targeting FOXC1 (PA1-807, Thermo Fisher Scientific Inc.). DNA fragments with a mean size of 200 bp were generated using sonication. The SYBR Green Master Mix from Bio-Rad and the primers listed in Table S5 were utilized for conducting real-time quantitative PCR [67–70].

### RNA-seq and polysome profiling

The TRIzol reagent (Invitrogen) was used to extract total RNA from 1 × 10^6^ tumor cells. To conduct polysome profiling, a sucrose density gradient of 15% to 45% (w/v) was newly made in an SW41 ultracentrifuge tube from Beckman (Indianapolis, IN). Cells were broken down in a solution containing polysome lysis buffer (100 mmol·l^−1^ KCl, 100 mg·ml^−1^ CHX, 2% Triton X-100, 5 mmol·l^−1^ MgCl_2_, and 10 mmol·l^−1^ HEPES, pH 7.4), and then placed on sucrose gradients for centrifugation at 36,000 × *g* for 2 h. A portion of each ribosome fraction was used to isolate total RNA using TRIzol reagent. Library creation and transcriptome sequencing were carried out using the Illumina HiSeq X Ten platform by Wuhan SeqHealth Technology Co., Ltd. (China). With the HTSeq v0.6.0 tool, 100-bp paired-end initial reads were aligned. The data were uploaded to Gene Expression Omnibus (GEO) under the accession numbers GSE294551 and GSE294552.

### Co-IP and proteomics analysis

The recombinant GST-tagged OGT and MBP-tagged FOXC1 proteins were prepared as previously described [67–70]. Preclearing lysates were undertaken by incubating with Protein A/G magnetic beads (HY-K0202, MedChemExpress) along with an isotype control [71]. Next, co-IP was performed as outlined in previous studies [67–70], using antibodies targeted toward FOXC1 (ab226219), OGT (ab177941), GST-tag (ab111947), HA-tag (ab1424), Flag-tag (ab125243, Abcam Inc.), or MBP-tag (66003-1-Ig, Proteintech Group, Inc., Rosemont, IL). The proteins attached to Protein A/G magnetic beads were analyzed using silver staining, Western blotting, and mass spectrometry (SpecAlly Life Technology Co., Ltd, Wuhan, China) [67–70].

### Immunofluorescence assay

The cells were placed on coverslips, treated with 4% paraformaldehyde, blocked by 5% milk for 1 h, and exposed to antibodies targeting FOXC1 (ab226219, Abcam Inc. 1:200 dilution), OGT (ab177941, Abcam Inc. 1:200 dilution), or OGT-570aa (ABclonal Biotechnology Co., Ltd, 1:200 dilution) overnight at 4 °C. Next, coverslips were exposed to Alexa Fluor 488-conjugated anti-rabbit IgG (4412) or Alexa Fluor 594-conjugated anti-mouse IgG (8890, Cell Signaling Technology Inc.), followed by DAPI (Sigma) staining. The photos were taken with a scanning confocal microscope from Nikon Instruments Inc, Japan [67–70].

### BiFC assay

The cDNA of human *FOXC1* or *OGT* was ligated into either pBiFC-VC155 or pBiFC-VN173 (Addgene). Recombinant plasmids were introduced into NB cells along with Xfect Transfection Reagent (631318, TaKaRa) through co-transfection for a period of 24 h. The confocal microscope (Biocompare, San Francisco, CA) was used to detect fluorescence with excitation (488 nm) and emission (500 nm) wavelengths [67–70].

### In vitro O-GlcNAcylation assay

Purified MBP-tagged FOXC1 (3 μg) was incubated with 5 μg of GST-tagged OGT or recombinant OGA protein (ab153117, Abcam Inc.) in 50 μl of reaction buffer (2 mmol·l^−1^ UDP-GlcNAc, 12.5 mmol·l^−1^ MgCl_2_, 1 mmol·l^−1^ DTT, and 50 mmol·l^−1^ Tris-HCl, pH 7.5) at 37 °C for 4 h, and then incubated with anti-MBP magnetic beads (MerckMillipore) at 4 °C for 3 h. The recovered proteins were analyzed via Western blotting.

### RIP assay

The ultraviolet light (254 nm, 200 J/cm^2^) was utilized for cellular cross-linking [68,69]. The EZ-Magna RIP RNA-Binding Protein Immunoprecipitation Kit (17-701, Sigma) was utilized for the RIP assay, along with antibodies targeting EIF4A3 (ab180573, Abcam Inc.). Recovered RNA was identified through real-time qRT-PCR using specific primers (Table S5).

### Assay for phase separation in vitro

To prepare recombinant proteins, a His-tagged *FOXC1* or *OGT* construct was transformed into BL21 strain (Biovector NTCC Inc., Beijing, China), then purified using Ni-NTA His-Tag Purification Agarose (HY-K0210, MedChemExpress). A phase separation test was conducted on dishes with glass bottoms. In a solution for droplet formation (10% glycerol, 50 mmol·l^−1^ Tris-HCl, and 1.0 mmol·l^−1^ dithiothreitol, pH 7.5), proteins (40 μmol·l^−1^) were mixed with crowding agent (10% polyethylene glycol 8000) and visualized using a Nikon A1R-SI confocal microscope with oil-immersion lenses [69,70].

### Assay for phase separation in vivo

Tumor cells growing on glass-bottomed dishes and expressing mCherry-tagged protein or endogenous protein were treated with Hoechst 33342 (HY-15559, MedChemExpress) for 10 min. Cells were observed with a Nikon A1R-SI confocal microscope after 2 PBS rinses. Visible phase separation puncta were identified as those with a diameter greater than 0.5 μm [69,70].

### FRAP test

For in vitro assay, droplets were exposed to 50% laser power (with 488- and 561-nm pulses) for 10 s, while a Nikon A1R-SI confocal microscope equipped with oil-immersion objectives captured time-series photos. The in vivo FRAP test was conducted with the utilization of a chamber for live-cell imaging. Laser pulses of 488 and 561 nm were employed to bleach droplets at 50% power for 5 s, followed by the observation of photobleaching recovery for 1 min. By using the FIJI/ImageJ (https://imagej.nih.gov/ij) program, fluorescence intensities were adjusted to match those before photobleaching occurred [69,70].

### Measurement of amino acids

To conduct flux experiments with [U-^13^C_5_] glutamine, cells were grown in glutamine-free medium (Sigma) with 10% FBS and 4 mmol·l^−1^ [U-^13^C_5_]-glutamine (Cambridge Isotope Labs, Tewksbury, MA) for 18 h. The metabolites were obtained by soaking with a mixture of methanol, acetonitrile, and water (in a ratio of 4.5:4.5:1) for 15 min, and then sonicated for 30 s at 4 °C. The liquid chromatography–mass spectrometry analysis was performed on the supernatants. Intracellular asparagine or alanine levels were measured by using the Asparagine Assay Kit (ab273333) or the L-Alanine Assay kit (ab83394, Abcam), respectively.

### Assay for protein synthesis

Tumor cells were treated by puromycin (10 μg·ml^−1^) for a duration of 15 min. Western blotting was used to detect puromycylated peptides with an antibody specific for puromycin (ab315887, Abcam Inc.). The Global Protein Synthesis Assay Kit (ab273286, Abcam Inc.) was used in the OP-Puro labeling assay to observe protein synthesis. Analysis of the percentage of positive cells was conducted to quantify newly synthesized OP-puro-labeled peptides [70].

### Measurement of GSH/GSSG, GPX4 activity, NADPH/NADP^+^, and liable iron

Intracellular GSH/GSSG levels, GPX4 activity, and NADPH/NADP^+^ ratio were determined by the GSH+GSSG/GSH Assay Kit (ab239709, Abcam), the GPX4 Activity Assay Kit (Elabscience, Houston, TX), or the NADPH/NADP Quantitation Kit (MAK038, Sigma). To monitor liable iron, cells were placed on coverslips, treated with RhoNox-1 (MedChemExpress), and dyed with DAPI (300 nmol·l^−1^, Sigma).

### Lipid peroxidation assay

Flow cytometry was conducted in accordance with previous documentation [72]. Cells were cultured with 1 μM C11-BODIPY (581/591) probe (RM02821, ABclonal Biotechnology Co., Ltd) for 1 h, and received 2 to 3 rinses with cold PBS. Next, flow cytometry was utilized to measure the fluorescence intensity of 1 × 10^4^ cells in each sample, with an excitation wavelength of 488 nm and an emission wavelength of 510 nm. The analysis of results was conducted with FlowJo software (BD Biosciences, Ashland, OR).

### Measurement of mitochondrial membrane potential

The tetraethylbenzimidazolyl carbocyanine iodide (JC-1) dye (HY-15534, MedChemExpress) was utilized for measuring the mitochondrial membrane potential.

### Transmission electron microscopy

On 10-cm dishes, tumor cells were plated and treated with 2.5% glutaraldehyde. Following a 2- to 3-h fixation with 1% osmic acid, cells were dried off and paraffin-embedded. Uranyl acetate-lead citrate was employed for staining sections that were 70 nm in thickness. Mitochondria were observed and photographed via transmission electron microscopy (Delong America Inc., Quebec, CA).

### Affinity purification

The FG beads from Nacalai Tesque, Inc (Kyoto, Japan) were mixed with 20 mmol·l^−1^ MN in *N*,*N*-dimethylformamide for incubation. Next, beads with MN immobilized (0.5 mg) were mixed with recombinant proteins and kept at 4 °C for 2 h. After that, proteins were washed with 0.5% NP-40 lysis buffer and analyzed using Western blotting [67].

### DSF assay

Recombinant GST-tagged OGT-570aa or MBP-tagged FOXC1 proteins were incubated with PBS containing SYPRO Orange dye (Invitrogen). Then, chemical compounds were added into the wells and heated from 40 °C to 90 °C (1 °C per minute), while fluorescence intensity was recorded at each 1 °C increment and graphed against temperature. The melting temperature (*T*_m_) of proteins was calculated using the Boltzmann equation [73].

### In vitro viability studies

For detecting in vitro viability, 3 × 10^3^ tumor cells were placed within 96-well plates, and cell viability was measured via the colorimetric assay with tetrazolium bromide (MTT, MerckMillipore) [68–70].

### In vitro growth assay

Tumor cells (5 × 10^3^ each well) were combined with 0.05% Nobel agar (Thermo Fisher Scientific Inc.), then placed on 6-well plates with solidified 0.1% Noble agar for 21 days of incubation. Cellular colonies were dyed with 0.5% crystal violet and then enumerated using a microscope [67–70].

### In vitro invasion assay

Cellular invasive ability was assessed using Matrigel matrix from BD Science (Sparks, MD) [67–70]. Tumor cells deprived of nutrients (1 × 10^5^ each well) were placed in the top compartment of a Transwell insert with 8.0-μm pores (Corning, New York, NY) for 24 h. Invaded cells were dyed using 0.1% crystal violet for a duration of 10 min and then examined under the microscope.

### Animal model

All animal studies followed the National Institutes of Health (NIH) Guidelines for the Care and Use of Laboratory Animals and were approved by Experimental Animal Ethics at Huazhong University of Science and Technology (approval number: 2021-3207). Four-week-old BALB/c nude mice were randomly selected for tumor formation or experimental metastasis studies [67–70]. In experiments involving treatment of live animals, tumor cells (1 × 10^6^ or 0.4 × 10^6^) that had been modified to produce red fluorescent protein were inserted into the dorsal flanks or tail vein of nude mice. Mice were randomly selected and treated with MN (50 mg/kg/day) through tail vein injection after 1 week, then imaged using the Pearl Impulse small animal imaging system (Licor Biosciences, Lincoln, NE) [67–70].

### Human tissue samples

The study of human tissues was granted by the Institutional Review Board of Union Hospital, Tongji Medical College (approval number: 2023-0519). All protocols followed were in line with the guidelines outlined in the Declaration of Helsinki. Every legal guardian of NB patients who had no preoperative chemotherapy or radiotherapy in the past signed a document giving their consent. From September 2023 to September 2024, tumor tissues were obtained during the operation, confirmed by a pathologist, and kept at −80 °C. Human dorsal root ganglia were obtained from terminated pregnancies.

### Immunohistochemistry

Antibodies targeting Ki-67 (PA5-19462, Thermo Fisher Scientific Inc.) or CD31 (MA3100, Thermo Fisher Scientific Inc.) were employed in immunohistochemical staining, and quantitative analysis was conducted as previously outlined [67–70].

### Statistical analysis

The average and standard error of the mean (SEM) were employed to illustrate the data. Cutoff values were established based on the average levels of gene expression. The Student’s *t* test or analysis of variance (ANOVA) was applied for comparing differences. Fisher’s exact test was used to determine the statistical significance of the overlap between 2 gene lists. The correlation coefficient by Pearson was utilized to investigate the connection of gene expression. The log-rank test was used to determine the difference in survival. Statistical tests were considered significant if false discovery rate-corrected *P* values were less than 0.05 in 2-sided tests.

## Data Availability

RNA-seq and polysome profiling data have been deposited in the Gene Expression Omnibus (GEO) database (https://www.ncbi.nlm.nih.gov/geo/, accession code: GSE294551 and GSE294552). Publicly available datasets can be found in the following: (i) the GEO database (GSE62564 and GSE209112, https://www.ncbi.nlm.nih.gov/geo); (ii) The Cancer Genome Atlas (TCGA, https://portal.gdc.cancer.gov); and (iii) the GSEA database (https://www.gsea-msigdb.org/gsea/index.jsp).
