# Supplementary material for: Targeting the Exonic Circular OGT RNA/O-GlcNAc Transferase/Forkhead Box C1 Axis Inhibits Asparagine- and Alanine-Mediated Ferroptosis Repression in Neuroblastoma Progression
Source: Research (Wash D C). 2025 May 23;8:0703. doi: 10.34133/research.0703 (PMC12099056; doi:10.34133/research.0703)
Supplement: Supplementary 1 — Figs. S1 to S12 Tables S1 to S7 [file research.0703.f1.pdf]

Supplementary Information

Targeting *ecircOGT*/*OGT*/*FOXC1* axis inhibits asparagine and alanine-mediated ferroptosis repression in neuroblastoma progression

Li et al.

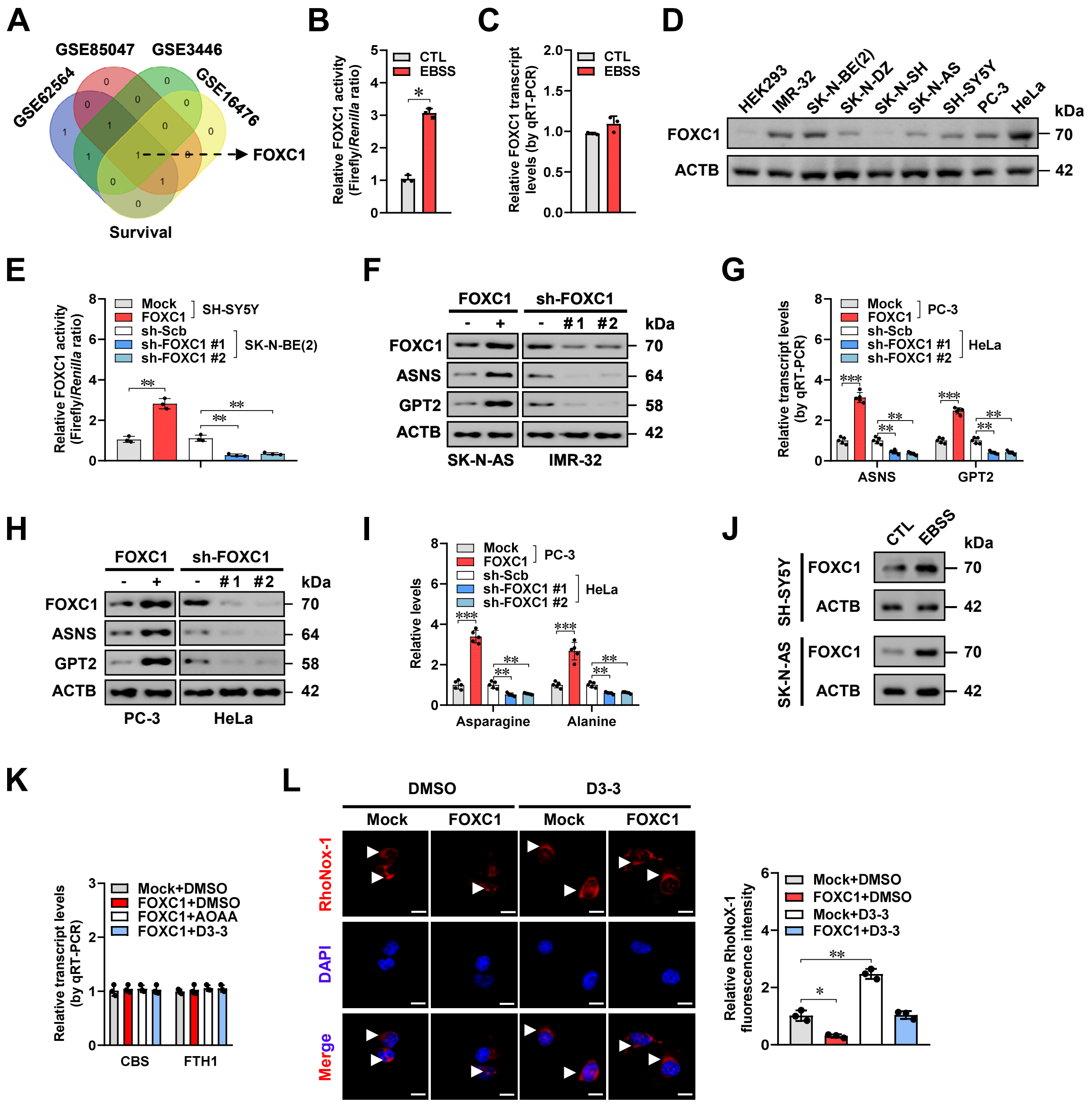

Figure S1. Expression profiles of *FOXC1* and its roles in repressing ferroptosis. A, Venn diagram indicating the association of identified transcriptional regulators of amino acid metabolic genes with survival of 498 (GSE62564), 283 (GSE85047), 102 (GSE3446), and 88 (GSE16476) NB patients. **B** and **C**, Dual-luciferase (B) and real-time qRT-PCR (C, normalized to *β-actin*) assays showing the activity and transcript levels of *FOXC1* in SH-SY5Y cells treated with control (CTL) DMEM/F12 or EBSS for 6 hours (n=3). **D**, Western blot assay showing the expression levels of FOXC1 in HEK293 cells, NB cell lines, PC-3 cells, or HeLa cells. **E**, Dual-luciferase reporter revealing the activity of FOXC1 in SH-SY5Y or SK-N-BE(2) cells stably transfected with empty vector (mock), *FOXC1*, scramble shRNA (sh-Scb), sh-FOXC1 #1, or sh-FOXC1 #2. **F**, Western blot assay revealing the expression levels of *ASNS* and *GPT2* in SK-N-AS or IMR-32 cells stably transfected with mock, *FOXC1*, sh-Scb, sh-FOXC1 #1, or sh-FOXC1 #2. **G** and **H**, Real-time qRT-PCR (G, normalized to *β-actin*, n=5) and western blot (H) assays showing the transcript and protein levels of *ASNS* and *GPT2* in PC-3 or HeLa cells stably transfected with mock, *FOXC1*, sh-Scb, sh-FOXC1 #1, or sh-FOXC1 #2. **I**, Relative asparagine and alanine levels in PC-3 or HeLa cells stably transfected with mock, *FOXC1*, sh-Scb, sh-FOXC1 #1, or sh-FOXC1 #2 (n=5). **J**, Western blot assay showing the FOXC1 protein expression in SH-SY5Y and SK-N-AS cells treated with DMEM/F12 (CTL) or EBSS for 6 hours. **K**, Real-time qRT-PCR assay indicating the transcript levels (normalized to *β-actin*) of *CBS* and *FTH1* in SH-SY5Y cells stably transfected with mock or *FOXC1*, and those treated with AOAA (1.0 μmol·L^{-1}) or D3-3 (10 μmol·L^{-1}, n=3). **L**, Representative images (left panel) and quantification (right panel) of RhoNox-1 staining in SH-SY5Y cells stably transfected with mock or *FOXC1*, and those treated with D3-3 (10 μmol·L^{-1}, n=3). Scale bars: 10 μm. Fisher's exact test for overlapping analysis in **A**. Student's t test or ANOVA compared the difference in **B**, **C**, **E**, **G**, **I**, **K** and **L**. * P<0.05, ** P<0.01, *** P<0.001. Data are shown as mean ± s.e.m. (error bars) or representative of three independent experiments in **B**-**L**.

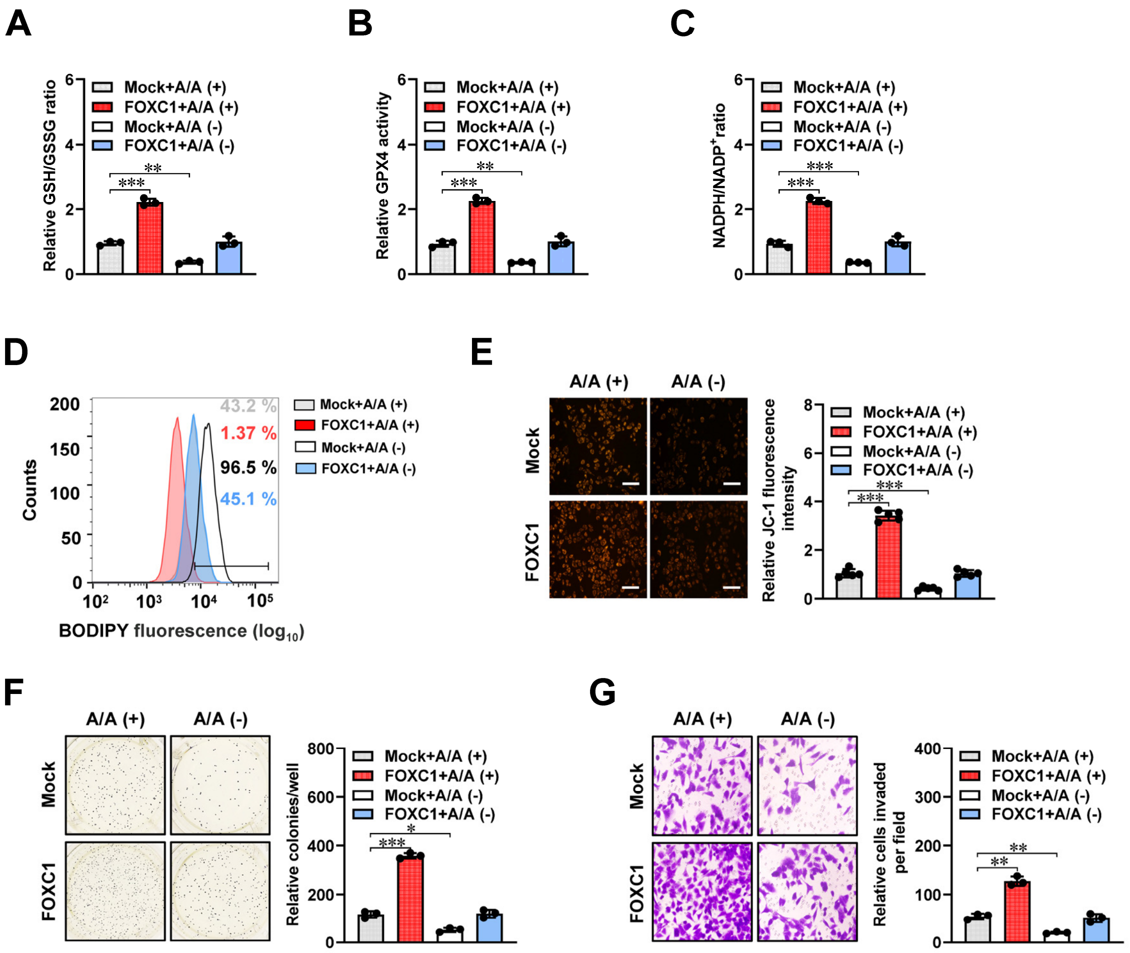

Figure S2. ***FOXC1* drives ferroptosis resistance, growth, and invasion of NB cells *in vitro*. A-C**, Relative GSH/GSSG ratio (A), GPX4 activity (B), and NADPH/$NADP^+$ ratio (C) in SH-SY5Y cells stably transfected with empty vector (mock) or *FOXC1*, and those treated with asparagine/alanine (A/A) supplement or deprivation (n=3). **D**, Flow cytometry showing the lipid ROS levels in SH-SY5Y cells stably transfected with mock or *FOXC1*, and those treated with A/A supplement or deprivation (n=3). **E**, Representative images of mitochondria shrunk in SH-SY5Y cells stably transfected with mock or *FOXC1*, and those treated with A/A supplement or deprivation. **F** and **G**, Representative images (left panel) and quantification (right panel) of soft agar (F) and matrigel invasion (G) in SH-SY5Y cells stably transfected with mock or *FOXC1*, and those treated with A/A supplement or deprivation (n=3). ANOVA compared the difference in **A-C** and **E-G**. * P<0.05, ** P<0.01, *** P<0.001. Data are shown as mean ± s.e.m. (error bars) or representative of three independent experiments in **A-G**.

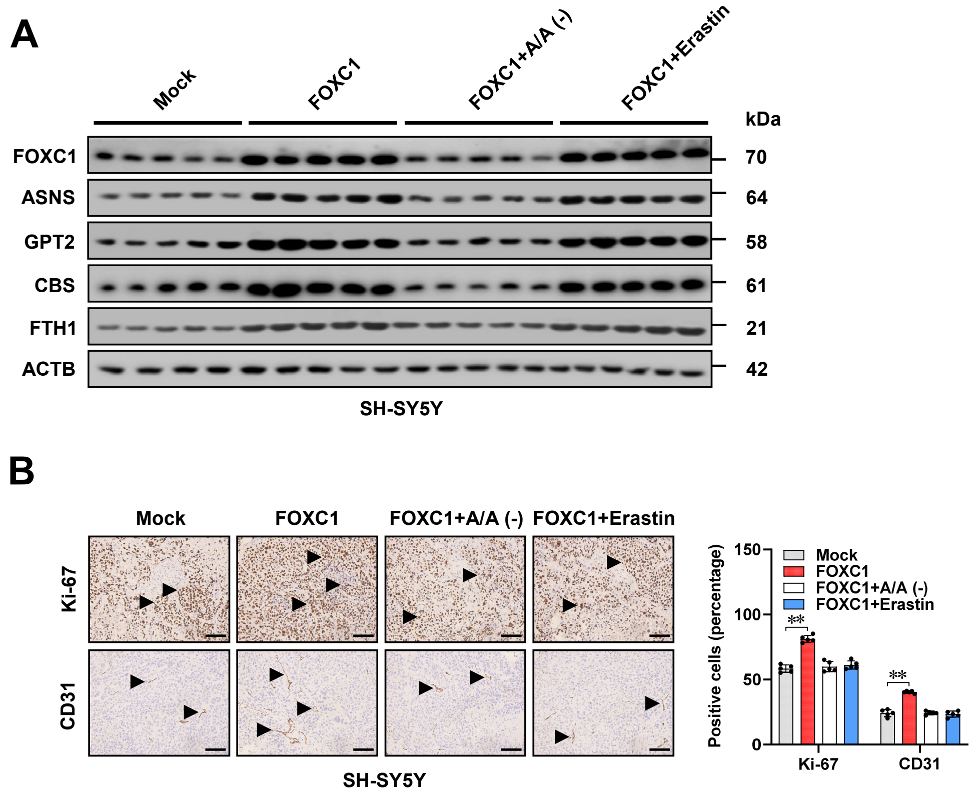

Figure S3. ***FOXC1*** **facilitates gene expression, proliferation, and angiogenesis of NB via facilitating asparagine and alanine biogenesis.** Western blot (A) and immunohistochemical staining (B) assays indicating the expression of downstream genes, Ki-67, and CD31 within subcutaneous xenografts in nude mice formed by SH-SY5Y cells stably transfected with empty vector (mock) or *FOXC1*, and those receiving asparagine/alanine (A/A)-free diet or erastin treatment (0.5 nmol·L^{-1} per mouse, n=5 for each group). Scale bars: 100 μm. ** P<0.01. Data are shown as mean ± s.e.m. (error bars) or representative of three independent experiments in **A** and **B**.

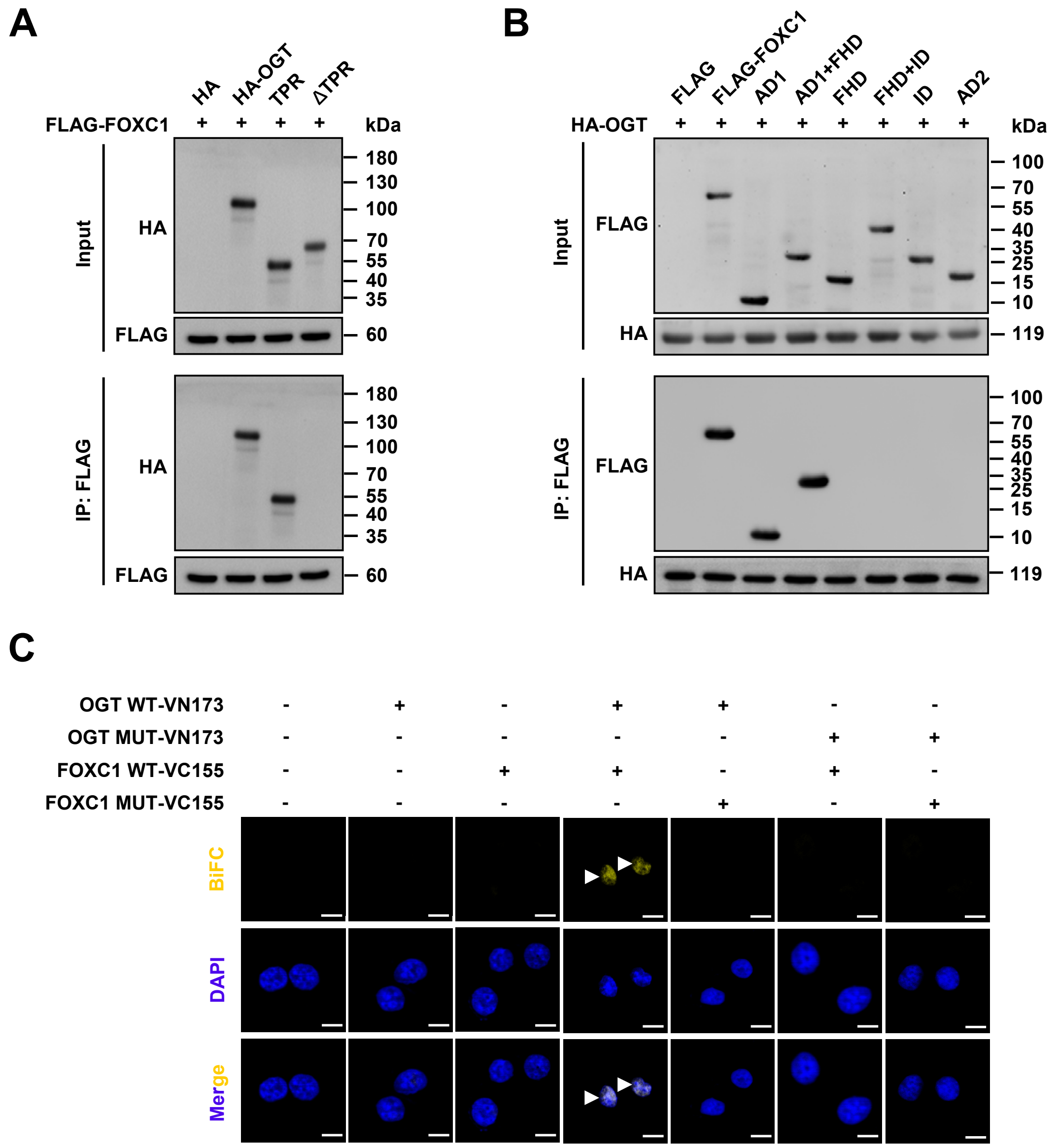

Figure S4. Interaction between OGT and FOXC1. A and **B**, Co-IP and western blot assays indicating the interaction between OGT and FOXC1 in SH-SY5Y cells transfected with wild-type or mutant HA-tagged *OGT* and FLAG-tagged *FOXC1* truncations. **C**, BiFC assay showing the interaction of OGT and FOXC1 in SH-SY5Y cells transfected with wild-type (WT) or mutant (Mut) pBiFC-VN173-OGT and pBiFC-VC155-FOXC1. Scale bars: 10 μm. Data are representative of three independent experiments in **A**-**C**.

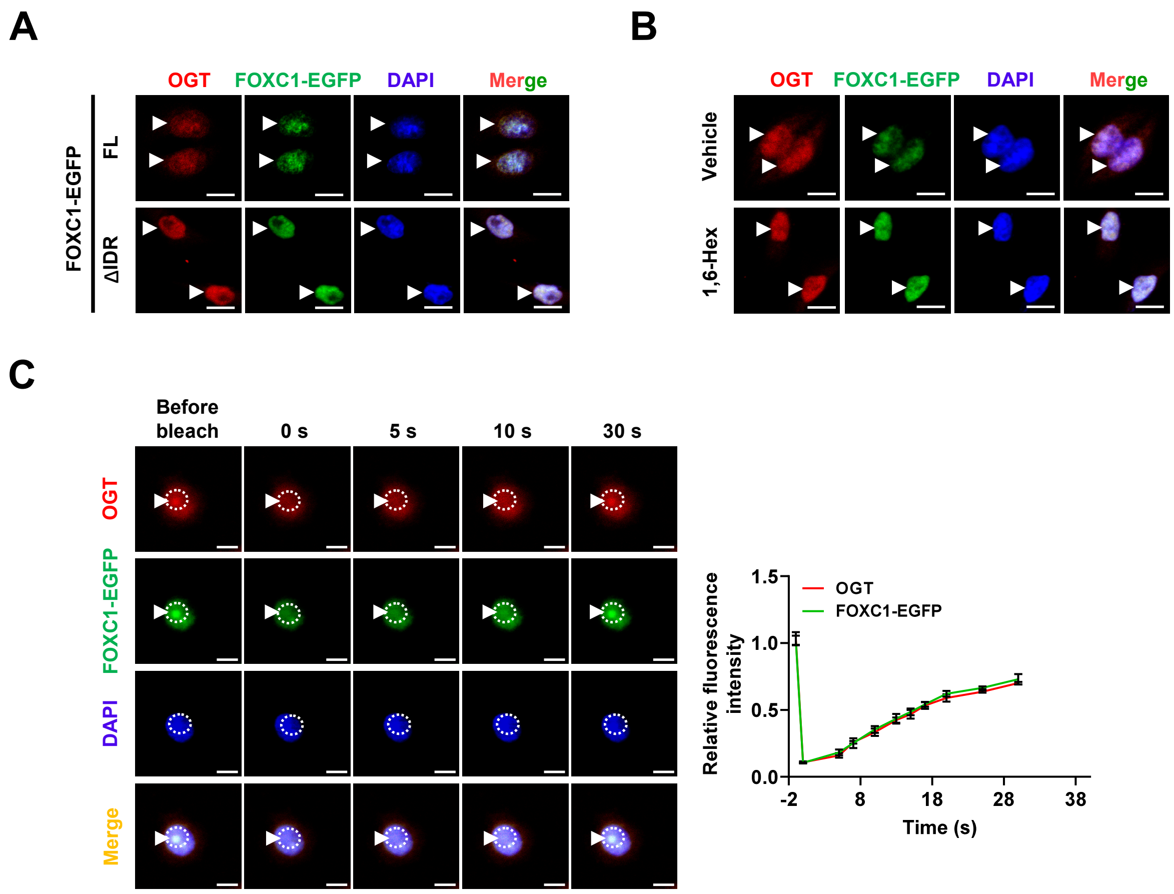

Figure S5. Phase separation of OGT and FOXC1. A and **B**, Fluorescence imaging assay showing the condensate formation of OGT and FOXC1-EGFP (arrowheads) in SK-N-AS cells transfected with full-length (FL) or IDR-deficient (ΔIDR) EGFP-tagged *FOXC1* (A), or those treated with 1.5% 1,6-hexanediol (1,6-Hex, B). **C**, Representative images (left panel) and quantification (right panel) of FRAP assay showing the exchange kinetics (arrowheads) of OGT and FOXC1-EGFP within condensates in SK-N-AS cells transfected with EGFP-tagged *FOXC1*. Scale bars: 10 μm. Data are shown as mean ± s.e.m. (error bars) or representative of three independent experiments in **A**-**C**.

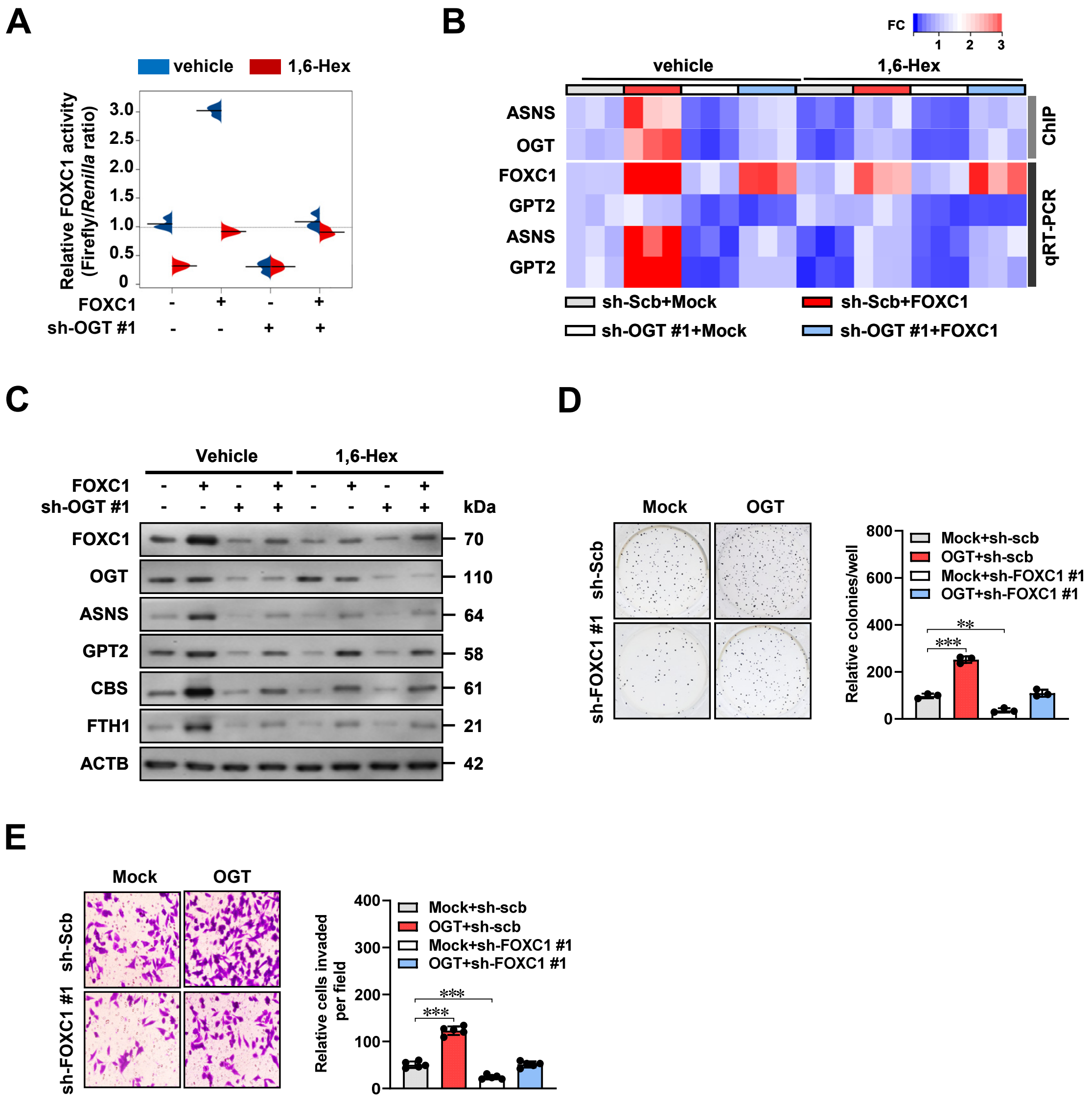

Figure S6. OGT cooperates with FOXC1 to facilitate gene expression, growth, and invasion of NB cells in a LLPS-dependent manner. A-C, Dual-luciferase (A, *n*=3), ChIP and qPCR (B, normalized to input, *n*=3), real-time qRT-PCR (B, normalized to *β-actin*, *n*=3), and western blot (C) assays showing the FOXC1 activity, FOXC1 enrichment, and downstream gene (*ASNS*, *GPT2*, *CBS*, and *FTH1*) expression in SK-N-AS cells stably transfected with empty vector (mock), *FOXC1*, scramble shRNA (sh-Scb), or sh-OGT #1. **D** and **E**, Representative images (left panel) and quantification (right panel) of soft agar (D) and matrigel invasion (E) in SK-N-BE(2) cells stably transfected with mock, *OGT*, sh-Scb, or sh-FOXC1 #1 (*n*=5). ANOVA compared the difference in **D** and **E**. ** P<0.01, *** P<0.001. Data are shown as mean ± s.e.m. (error bars) or representative of three independent experiments in **A**-**E**.

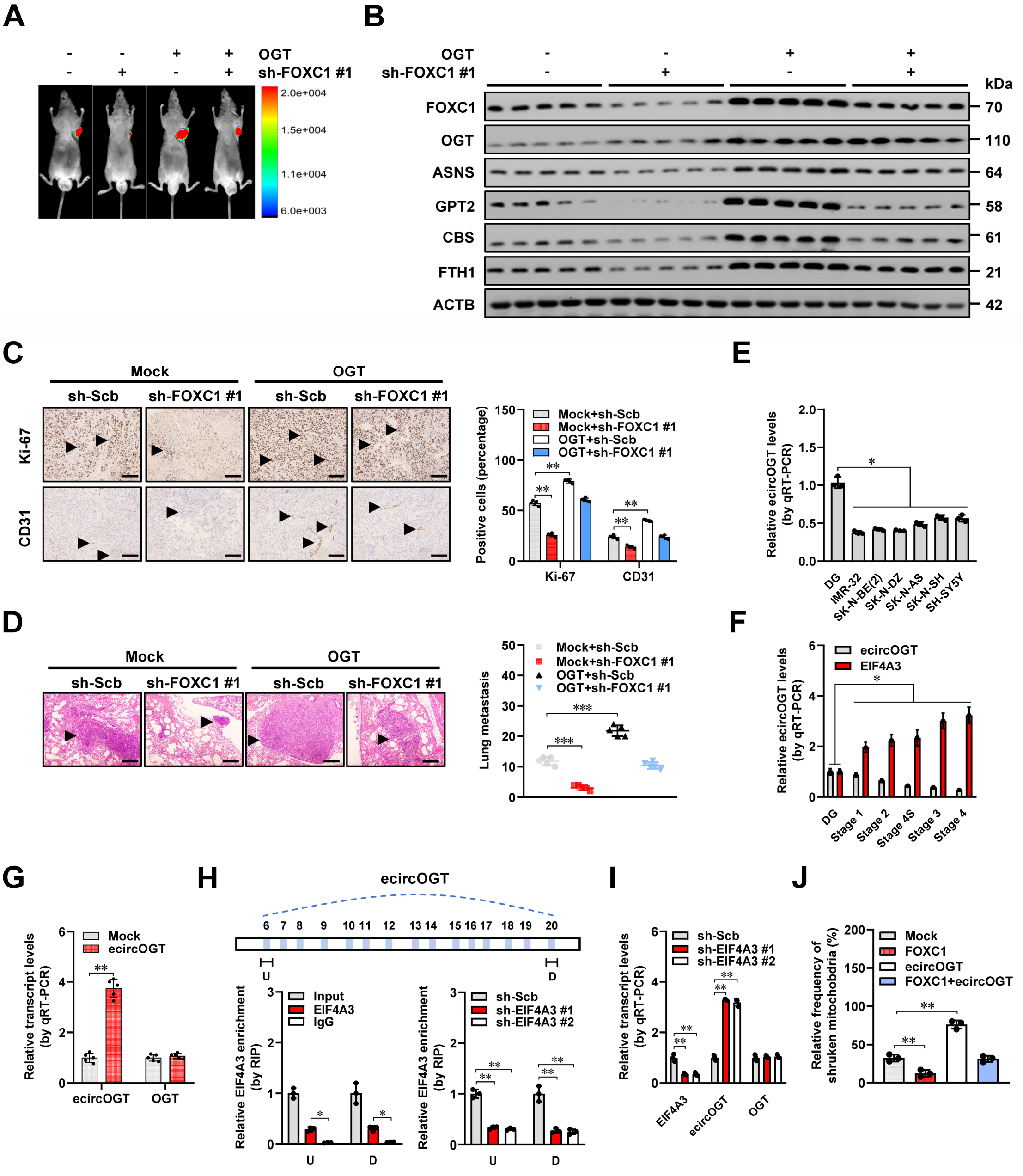

Figure S7. OGT cooperates with FOXC1 to facilitate tumorigenesis and aggressiveness. A-C, *In vivo* imaging (A), western blot (B), and immunohistochemical staining (C) indicating the growth, gene expression, and Ki-67 or CD31 levels within subcutaneous xenografts in nude mice formed by SK-N-BE(2) cells stably transfected with empty vector (mock), *OGT*, scramble shRNA (sh-Scb), or sh-FOXC1 #1 (*n*=5 for each group). Scale bars: 100 μm. **D**, HE staining showing the lung metastasis in nude mice treated with subcutaneous or tail vein injection of SK-N-BE(2) cells stably transfected with mock, *OGT*, sh-Scb, or sh-FOXC1 #1 (*n*=5 for each group). **E** and **F**, Real-time qRT-PCR showing the levels (normalized to *β-actin*) of *ecircOGT* and *EIF4A3* in normal dorsal root ganglia (DG, *n*=3), cultured NB cell lines (E), and NB tissues of different INSS stages (F, *n*=15). **G**, Real-time qRT-PCR showing the levels (normalized to *β-actin*) of *ecircOGT* and *OGT* in SK-N-BE(2) cells stably transfected with mock or *ecircOGT* (*n*=5). **H**, RIP assay indicating endogenous enrichment of EIF4A3 on upstream (U) or downstream (D) flanking regions of *ecircOGT* in SH-SY5Y cells, and those transfected with sh-Scb, sh-EIF4A3 #1, or sh-EIF4A3 #2 (*n*=3). **I**, Real-time qRT-PCR showing the levels (normalized to *β-actin*) of *EIF4A3*, *ecircOGT*, and *OGT* in SH-SY5Y cells stably transfected with sh-Scb, sh-EIF4A3 #1, or sh-EIF4A3 #2 (*n*=3). **J**, Quantification of mitochondria shrunk in SK-N-BE(2) cells stably transfected with mock, *FOXC1*, or *ecircOGT* (*n*=3). ANOVA compared the difference in **C**-**J**. * *P*<0.05, ** *P*<0.01, *** *P*<0.001. Data are shown as mean ± s.e.m. (error bars) or representative of three independent experiments in **A**-**J**.

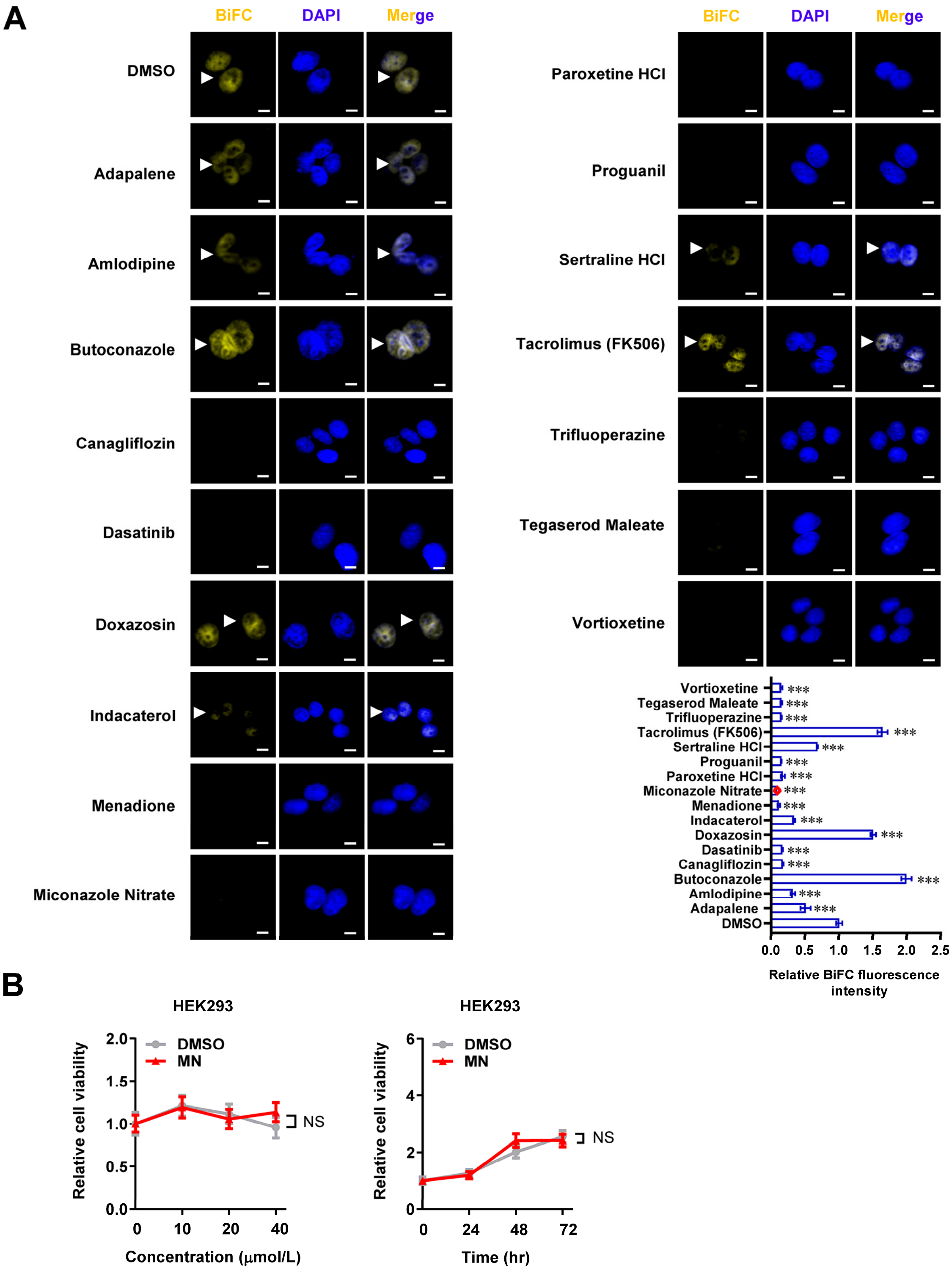

Figure S8. Screening of inhibitors targeting OGT-FOXC1 interaction. A, Representative images and quantification of BiFC assay showing the effects of chemicals on physical interaction of OGT and FOXC1 in SK-N-BE(2) cells transfected with pBiFC-VN173-OGT and pBiFC-VC155-FOXC1. Scale bars: 10 μm. **B**, MTT colorimetric assay indicating the viabilities of HEK293 cells treated with DMSO or MN for 48 hours, or MN (20 $\mu mol \cdot L^{-1}$) for duration as indicated. Student's *t* test or ANOVA compared the difference in **A** and **B**. *** *P*<0.001. NS, non-significant. Data are shown as mean ± s.e.m. (error bars) or representative of three independent experiments in **A** and **B**.

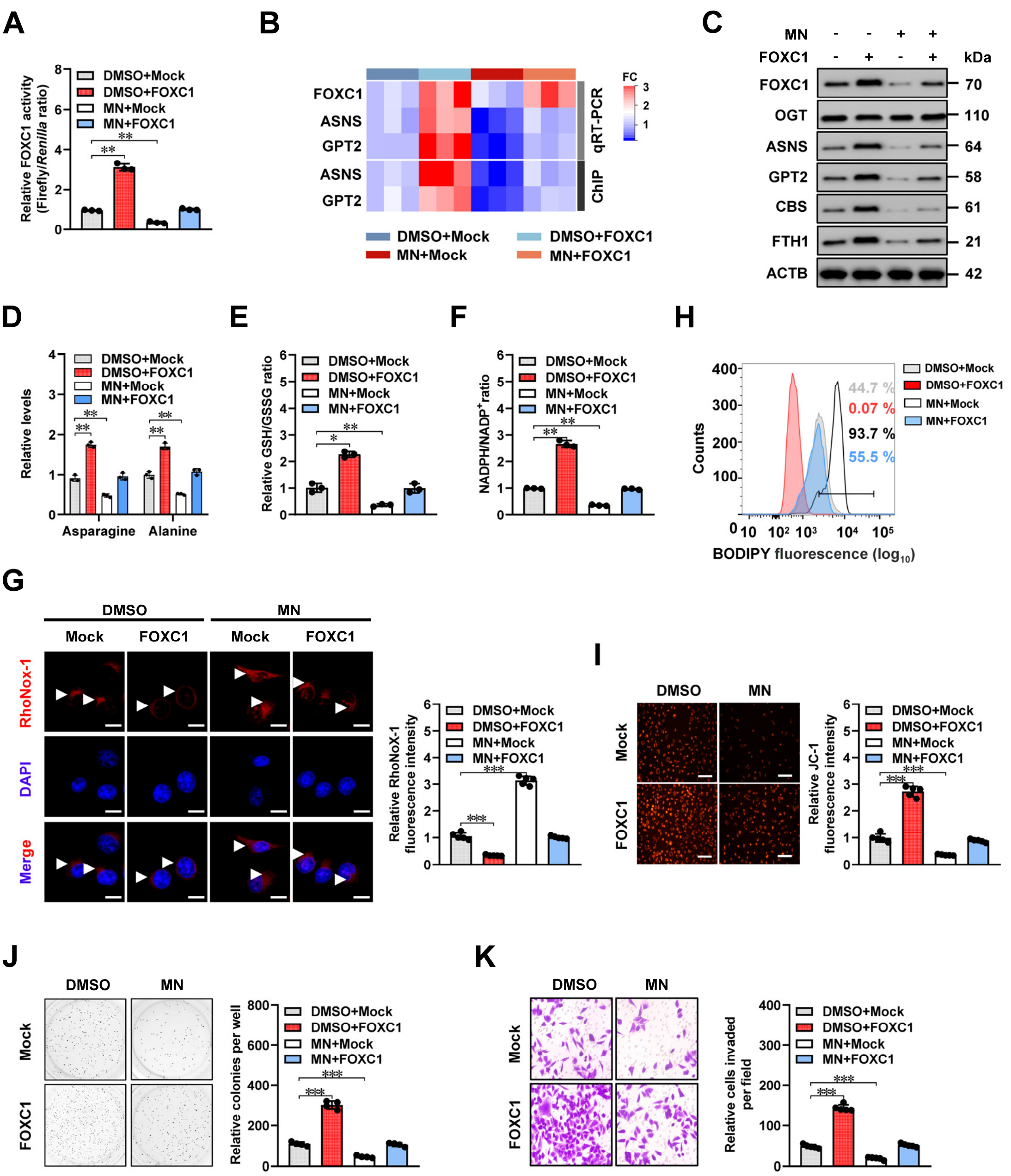

Figure S9. MN induces ferroptosis of NB cells via repressing FOXC1 activity. A-C, Dual-luciferase (A), ChIP and qPCR (B, normalized to input), real-time qRT-PCR (B, normalized to *β-actin*), and western blot (C) assays showing the FOXC1 activity, FOXC1 enrichment, and downstream gene (*ASNS*, *GPT2*, *CBS*, and *FTH1*) expression in SK-N-BE(2) cells treated with DMSO or MN (20 μmol·L^{-1}), and those transfected with *FOXC1* (*n*=3). **D-F**, Relative asparagine and alanine levels (D), GSH/GSSG ratio (E), and NADPH/NADP^{+} ratio (F) in SK-N-BE(2) cells treated with DMSO or MN (20 μmol·L^{-1}), and those transfected with *FOXC1* (*n*=3). **G**, Representative images (left panel) and quantification (right panel) of RhoNox-1 staining in SK-N-BE(2) cells treated with DMSO or MN (20 μmol·L^{-1}), and those transfected with *FOXC1* (*n*=3). Scale bars: 10 μm. **H**, Flow cytometry showing the lipid ROS levels in SK-N-BE(2) cells treated with DMSO or MN (20 μmol·L^{-1}), and those transfected with *FOXC1* (*n*=3). **I**, Representative images (left panel) and quantification (right panel) of JC-1 (2 μg/ml) staining in SK-N-BE(2) cells treated with DMSO or MN (20 μmol·L^{-1}), and those transfected with *FOXC1* (*n*=3). **J** and **K**, Representative images (left panel) and quantification (right panel) of soft agar (J) and matrigel invasion (K) in SK-N-BE(2) cells treated with DMSO or MN (20 μmol·L^{-1}), and those transfected with *FOXC1* (*n*=3). ANOVA compared the difference in **A**, **D-F**, and **G-K**. * *P*<0.05, ** *P*<0.01, *** *P*<0.001. Data are shown as mean ± s.e.m. (error bars) or representative of three independent experiments in **A-K**.

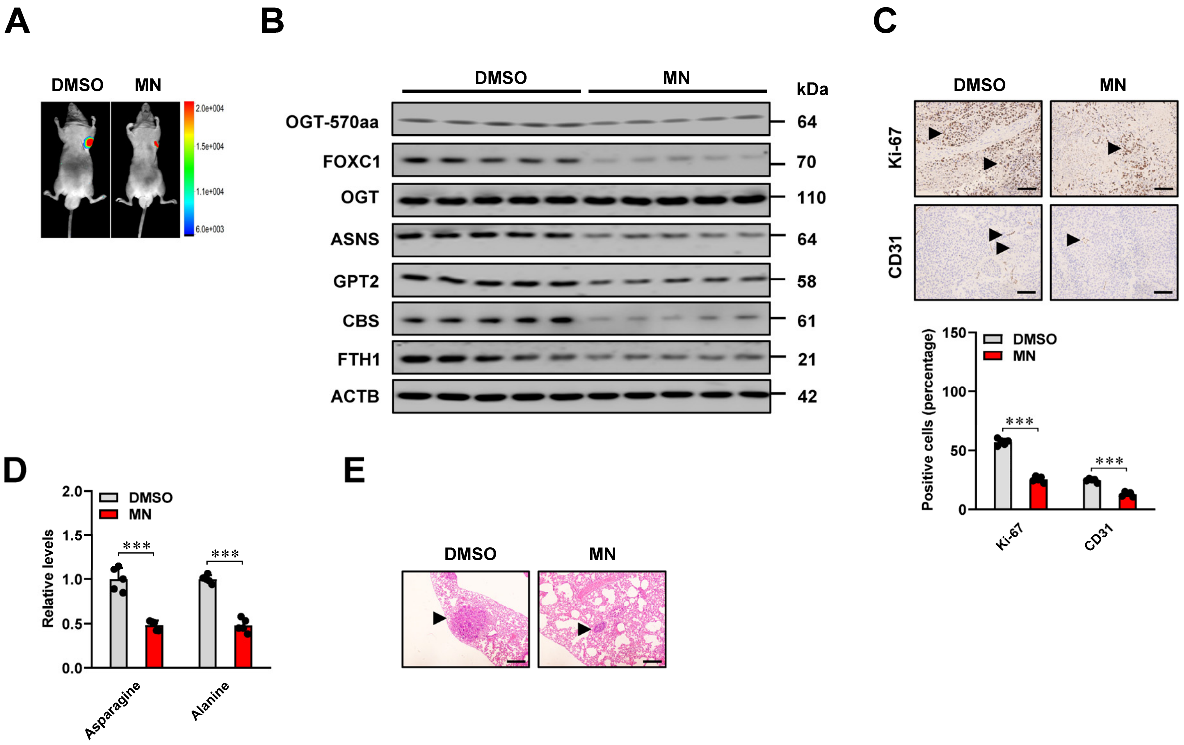

Figure S10. MN inhibits tumorigenesis and aggressiveness *in vivo*. A-C, *In vivo* imaging (A), western blot (B), and immunohistochemical staining (C) assays indicating the growth, gene expression, and Ki-67 or CD31 levels within subcutaneous xenografts in nude mice formed by SK-N-BE(2) cells, those treated with DMSO or MN (50 mg·kg^{-1}, n=5 for each group). Scale bars: 100 μm. **D**, Relative aspartate and asparagine levels in subcutaneous xenografts in nude mice formed by SK-N-BE(2) cells, those treated with DMSO or MN (50 mg·kg^{-1}, n=5 for each group). **E**, HE staining showing the lung metastasis in nude mice treated with subcutaneous or tail vein injection of SK-N-BE(2) cells, and treated with DMSO or MN (50 mg·kg^{-1}, n=5 for each group). Student's t test compared the difference in **C** and **D**. *** P<0.001. Data are shown as mean ± s.e.m. (error bars) or representative of three independent experiments in **A-E**.

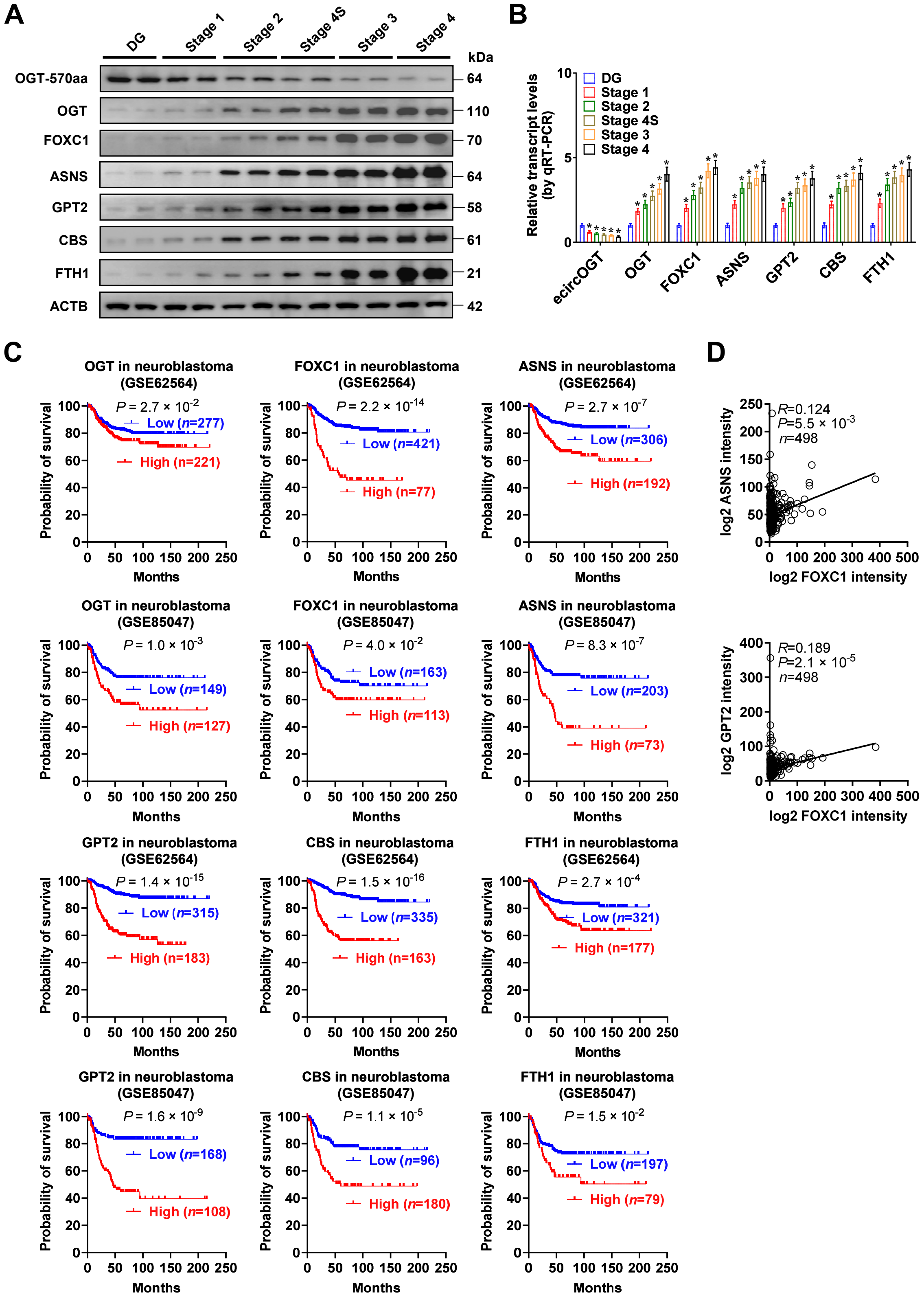

Figure S11. Expression of *OGT-570aa*, *OGT*, *FOXC1*, and downstream genes in NB. A, Western blot showing the expression of OGT-570aa, OGT, FOXC1, ASNS, GPT2, CBS, or FTH1 in normal dorsal ganglia (DG) and NB tissues with different INSS stages. **B**, Real-time qRT-PCR assay revealing the transcript levels (normalized to *β-actin*) of *ecircOGT*, *OGT*, *FOXC1*, *ASNS*, *GPT2*, *CBS*, or *FTH1* in DG (*n*=3) and NB tissues with different INSS stages (*n*=15). **C**, Kaplan-Meier curves indicating the survival of 498 (GSE62564) and 283 (GSE85047) NB patients with low or high expression of *OGT* (cutoff values=7.49 and 9.07), *FOXC1* (cutoff values=3.52 and 6.84), *ASNS* (cutoff values=5.62 and 8.35), *GPT2* (cutoff values=5.21 and 6.44), *CBS* (cutoff values=5.89 and 7.58), or *FTH1* (cutoff values=8.47 and 5.72). **D**, Expression correlation of *FOXC1* with that of *ASNS* or *GPT2* in 498 NB patients (GSE62564). ANOVA compared the difference in **B**. Log-rank test for survival comparison in **C**. Pearson's correlation coefficient assay in **D**. * *P*<0.05 vs. DG. Data are shown as mean ± s.e.m. (error bars) or representative of three independent experiments in **A** and **B**.

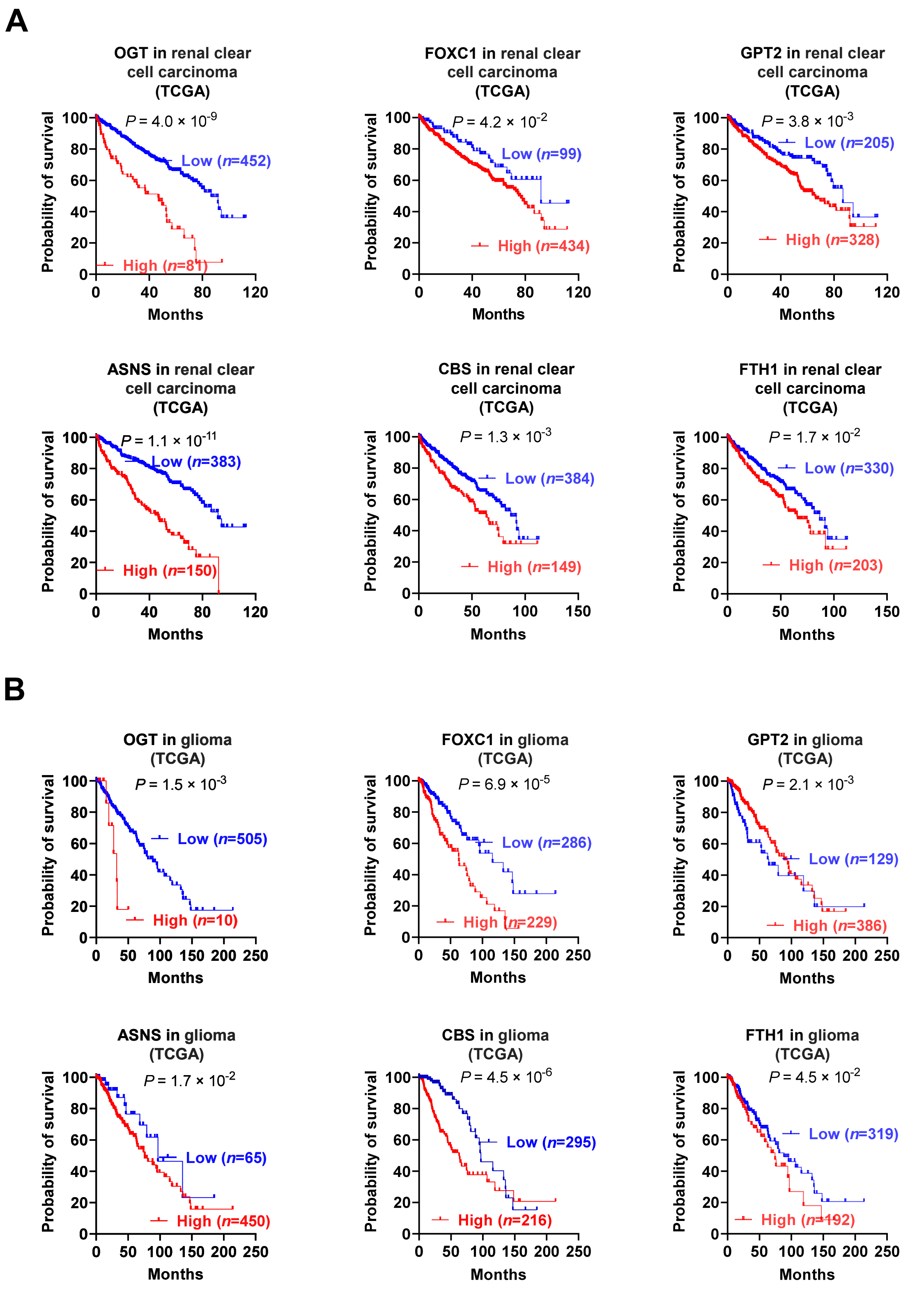

Figure S12. Kaplan-Meier curves of *OGT*, *FOXC1*, and downstream genes in multiple cancers. Kaplan-Meier curves indicating the survival of renal clear cell carcinoma or glioma patients with low or high expression of *OGT*, *FOXC1*, *ASNS*, *GPT2*, *CBS*, or *FTH1*. Log-rank test for survival comparison.

Table S1 Transcription factors of amino acid (AA) metabolic genes

AA metabolic genes	Transcription factors								
AA (-) vs. AA (+)	AA (-) vs. AA (+)								
ASNS	AHR	E2F1	GATA2	JUND	NFE2	PITX1	RXRA	STAT1	TP53
CBS	APEX1	E2F6	GATA3	KLF13	NFIA	PITX2	SAMD9L	STAT3	USF1
GCLM	ARNT	ELK1	GFI1	KLF5	NFIC	PLAU	SMAD4	STAT5B	USF2
GPT2	ATF2	ELK4	GLI2	LEF1	NFKB1	POU1F1	SMARCA2	TBP	WT1
HGD	ATF4	ETS1	HIF1A	MAX	NFYA	POU2F1	SNAI1	TCF3	YY1
KMO	BRCA1	ETV4	HINFP	MEF2A	NR1I2	POU2F2	SNAI2	TCF4	ZBTB7A
MCCC2	CACYBP	FOS	HNF1A	MIB2	NR2C2	PPARG	SP1	TCFAP2A	ZFHX3
MPST	CBEPA	FOXA1	HNF4A	MIR133B	NR2F1	PRDM1	SP3	TEAD2	ZNF148
NAT8L	CBEPB	FOXC1	HOXA5	MIR138	NR3C1	RARA	SP4	TEAD4	ZNF281
P4HA2	CEBPB	FOXF1	HOXD9	MTF1	NR5A1	RBPJ	SPI1	TFAP2A	ZNF354C
PCCA	CEBPD	FOXJ1	IRF7	MYB	NR5A2	RELA	SPIB	TFAP2C	
PIPOX	CEBPE	FOXL1	IRF8	MYC	PCBP1	RELB	SREBF1	TFAP2D	
SAT2	CREB1	FOXO3A	JDP2	MYOG	PDX1	RORB	SREBF2	THRA	
TPO	CRTC1	GATA1	JUN	MZF1	PGR	RUNX1	SRF	THRB	

Table S2 Polysome profiling of translating mRNAs increased by asparagine and alanine

AARS	C11orf74	DDIT3	GCLM	IPO5P1	NEO1	PLEKHH3	RP11-218M22.1	RP5-850E9.3	TMCC1-AS1
ABCC3	C15orf52	DDR2	GDF15	IQCH-AS1	NEURL1B	PLSCR4	RP11-21L23.4	RPL23AP87	TMEFF1
ABCG1	C17orf67	DGKG	GFOD1	IRF2	NFIL3	PMAIP1	RP11-227H15.5	SARS	TMEM37
ABCG2	C20orf196	DHRS2	GID4	IRX3	NIPAL2	PNRC1	RP11-230F18.6	SATB2-AS1	TMEM59L
AC000068.9	C9orf91	DHRS7	GKAP1	ISL2	NKX3-1	POPDC3	RP11-262H14.3	SBSN	TMIE
AC002401.1	CACNA1D	DICER1-AS1	GLIDR	ITPKB	NLRP1	POU6F1	RP11-262H14.4	SCIMP	TMOD2
AC007362.3	CAPN5	DKFZP434H168	GLIPR2	JAKMIP3	NME9	PPARGC1B	RP11-284F21.7	SDSL	TNFRSF25
AC007405.6	CARS	DLGAP1-AS2	GLIS3-AS1	JMY	NNMT	PPM1H	RP11-284F21.9	SEL1L3	TRANK1
AC008522.1	CASP16P	DMGDH	GLT8D2	JUNB	NOL4L	PPM1K	RP1-130H16.18	SEMA4C	TRIB3
AC009228.1	CBS	DNAH3	GLTSCR1L	KCNJ9	NPHS1	PPP1R9B	RP11-319G6.1	SEMA6D	TRIM16
AC009229.6	CBSL	DNAJB13	GNAO1	KCNN1	NPTXR	PRKCE	RP11-326C3.11	SERPINB9P1	TRIM16L
AC009542.2	CBX4	DNAJC18	GNAZ	KCTD1	NQO1	PRKD2	RP11-333E1.1	SESN2	TRIM2
AC011738.4	CCDC13	DNAJC27-AS1	GPAT2	KIAA0319	NR1D1	PRSS16	RP11-352G18.2	SH2B3	TRIM62
AC079922.3	CCDC146	DOPEY2	GPAT3	KIAA1211	NR4A2	PRX	RP11-353N14.7	SH3KBP1	TRIM66
AC104534.2	CCDC187	DUOX1	GPR1	KIAA1456	NRP2	PSAT1	RP11-356J5.12	SH3RF3	TRIM74
AC116609.2	CCT6B	DUSP6	GPR20	KIF21B	NUP50-AS1	PSMG3-AS1	RP11-359B12.2	SHC3	TSGA10
AC139099.5	CD177	ECM2	GPR3	KIF26B	NUPR1	PSPH	RP11-37B2.1	SHOX2	TSHZ1
AC139099.6	CD22	EGFL8	GRAMD1B	KLF6	NUTM2A	PTCH1	RP11-384P7.7	SIPA1L2	TSPAN33
AC240274.1	CDRT1	EGR1	GRB7	KLF9	NUTM2D	PTGES2-AS1	RP11-385D13.1	SIX1	TSSK3
AC241377.1	CDYL2	EIF4EBP1	GREB1	KLHDC9	OASL	PTK2B	RP11-392P7.6	SIX4	TULP4
ACTA2	CFAP70	ELFN1	GSDMB	KRT81	OR2A20P	PTPRJ	RP11-395G23.3	SLC12A8	ULBP1
ACTA2-AS1	CGREF1	ELOVL3	GTPBP2	KRTAP5-1	ORAI1	PXK	RP11-417N10.4	SLC1A5	USP27X
ACVR2B	CH17-270A2.1	ENOX2	HAP1	LAMP3	ORAI3	QRSL1	RP11-428J1.4	SLC22A15	UST
ADM2	CH17-472G23.2	ERFE	HCG27	LARP6	OSBPL6	RAB3A	RP11-434E6.5	SLC22A18	VEPH1
AF127936.7	CHAD	ERLEC1P1	HCP5	LCA5	OSGIN1	RASGEF1B	RP11-435O5.5	SLC22A18AS	VGF
AGER	CHN2	ERN1	HDAC9	LCN10	PAPPA	RBM26-AS1	RP11-43F13.3	SLC22A23	WARS
AJ003147.8	CHRM3	ETV5	HECW1	LCN2	PAPPA2	RBM43	RP11-441O15.3	SLC26A10	WDR7
ALDH1L2	CHRNB1	EXTL2	HHEX	LGSN	PAPPA-AS1	RFESD	RP11-443P15.2	SLC47A1	XPOT
ALPK3	CISH	EYA4	HINFP	LINC00623	PARD6B	RFX3	RP11-445F12.1	SLC48A1	XPOTP1
AMDHD1	CKMT2-AS1	FALEC	HIST1H1E	LINC00662	PAX6	RGMB	RP11-452K12.4	SLC6A7	XXbac-BPG181B23.6
ANGPTL4	CLCN4	FAM120C	HIST1H2BH	LINC00847	PCDH1	RGS14	RP11-474O21.5	SLC7A11	XXbac-BPGBPG55C20.2
ANKRA2	CLCN5	FAM129A	HIST2H2BF	LINC00987	PCDHA1	RHBDD1	RP11-47I22.4	SLC7A11-AS1	YARS
ANKRD29	CLDN23	FAM131A	HKDC1	LINC01133	PCDHA10	RHEBL1	RP11-495P10.10	SLFN5	ZBED3-AS1
ANKRD34A	CLDN4	FAM160B1	HLCS	LINC01137	PCDHA11	RILPL1	RP11-495P10.5	SNAI1	ZBTB18
AP000472.2	CLGN	FAM167B	HMGA1P4	LINC01356	PCDHA12	RNF165	RP11-495P10.7	SNORD15A	ZBTB46
AP001347.6	CLUHP3	FAM214B	HMGB1P31	LINC01484	PCDHA13	RNF187	RP11-495P10.9	SNORD5	ZBTB7B
AP006222.2	COL25A1	FAM229A	HNF4G	LINC01569	PCDHA2	RNFT2	RP11-496H1.2	SPRY4	ZC3H12A
APLF	COMT	FAM27E2	HOMER2	LIPH	PCDHA3	RNU7-1	RP11-498C9.12	SPSB2	ZC3H12D
APOL6	CPAMD8	FAM27E3	HOMEZ	LMX1B	PCDHA4	RNY3P15	RP11-523H20.3	SPX	ZDHHC14
ARHGAP31	CPEB3	FBXO10	HOXA1	LRIG3	PCDHA5	ROR1	RP11-539L10.2	SRXN1	ZEB1-AS1
ARHGEF2	CREBRF	FBXW10	HOXA3	LRRK2	PCDHA6	RP1-102K2.8	RP11-53O19.1	SSH2	ZFP36
ARHGEF37	CRHR1-IT1	FGF9	HOXA6	LSM10	PCDHA7	RP11-1000B6.5	RP11-54O7.10	STOX1	ZFP64
ARID1B	CRYM-AS1	FIGN	HOXA7	LUCAT1	PCDHA8	RP11-104G3.2	RP11-600F24.7	STX11	ZFPM2-AS1
ARL4D	CSF2RA	FKRP	HOXA-AS2	MAFG	PCDHA9	RP11-104G3.7	RP11-632F7.3	STXBP5L	ZHX2
ASNS	CTB-55O6.12	FLJ12825	HOXA-AS3	MAP1B	PCDHAC1	RP11-1109F11.3	RP11-638I2.8	SULF1	ZIC2
ASPDH	CTC-277H1.7	FOS	HOXB3	MAPRE3	PCGF1	RP11-110G21.1	RP11-723O4.2	SYN2	ZKSCAN8
ATP6AP1L	CTC-281F24.1	FOXL2NB	HOXB6	MARS	PCK2	RP11-115C10.1	RP11-797H7.1	SYT17	ZMYND15
ATXN7L1	CTC-338M12.6	FOXP2	HOXB8	MAST3	PCLO	RP11-118B22.4	RP11-823E8.3	TBC1D10C	ZNF217
B4GALT6	CTC-497E21.5	FRAT1	HOXB9	MEF2B	PCMTD1	RP11-1275H24.1	RP11-834C11.14	TBCEL	ZNF250
BCAS4	CTC-573N18.1	FRAT2	HOXB-AS1	MEGF8	PCNX2	RP11-12A20.10	RP11-933H2.4	TBKBP1	ZNF252P-AS1
BCL2	CTD-2036P10.3	FRS3	HOXB-AS3	MEX3A	PDE7B	RP11-165J3.6	RP1-225E12.2	TBX2	ZNF425
BCL6	CTD-2139B15.5	FTH1	HSD17B1	MIR1199	PELI1	RP11-166P13.3	RP13-143G15.4	TCEA1	ZNF555
BCL7A	CTD-2192J16.17	FUT10	HSD17B1P1	MIR99AHG	PER1	RP11-167N4.2	RP13-20L14.10	TCEA1P2	ZNF609
BCORL1	CTD-2265O21.7	FUT8	IDH1	MLLT4	PEX11A	RP11-178C3.2	RP13-516M14.1	TEC	ZNF627
BEND6	CTD-2377D24.6	FZD10-AS1	IDNK	MOCOS	PFN4	RP11-190A12.8	RP13-977J11.2	TFAP4	ZNF804A
BICDL1	CTD-2529O21.1	FZD7	IFI35	MURC	PHGDH	RP11-196G18.22	RP1-69M21.2	TGFA	ZNF815P
BMPR1B	CTD-2619J13.19	GABPB1-AS1	IFIH1	MVB12B	PIK3IP1	RP11-196G18.24	RP1L1	TGFB3	ZNF821
BNC2	CTD-3199J23.6	GABPB2	IFIT2	MX1	PIM1	RP11-203L2.4	RP3-522D1.1	THRB	ZNF862
BRD3	CYP11A1	GABRA3	IFNLR1	N4BP3	PIP5K1B	RP11-206M11.7	RP4-751H13.7	TIMP4	ZSCAN2
BSN	CYP2F2P	GALNT13	IGFN1	NBPF1	PIP5KL1	RP11-20D14.6	RP5-1050D4.3	TLCD2	ZSWIM5
BSND	CYP2U1	GARS	IGSF1	NBR2	PLA2G4C	RP11-213H15.4	RP5-1087E8.3	TLE3	
C10orf91	DAB2IP	GAS5	INHBE	NCKAP5L	PLAG1	RP11-216L13.16	RP5-1187M17.10	TLN2	

Table S3 Polysome profiling of translating mRNAs decreased by asparagine and alanine

3-Mar	C12orf75	CTB-49A3.2	FJX1	IQCC	MYADM	RND1	RP11-752L20.3	SRSF2	ZNF565
A2ML1-AS1	C16orf71	CTB-50L17.8	FN1	ITGA2B	MYH16	RNF217-AS1	RP11-839G9.1	SRSF3	ZNF587B
AC005822.1	C17orf53	CTB-76P12.1	FOSB	JAM3	MYO1A	RP11-10C24.1	RP11-876N24.5	SRSF6	ZNF625-ZNF20
AC006547.8	C1orf106	CTC-428G20.6	FOXC1	JMJD1C-AS1	NAT1	RP11-10C24.3	RP13-672B3.2	SRSF7	ZNF788
AC008440.5	C1orf116	CTC-490G23.2	FOXF2	JUND	NCOA5	RP11-1148L6.9	RP1-40E16.12	SSTR1	ZNF799
AC009121.1	C1QL1	CTD-2012J19.3	FOXQ1	KB-1732A1.1	NDRG1	RP11-116N8.4	RP1-63G5.7	STK17A	ZNF860
AC010184.1	C2orf16	CTD-2033D15.1	FSIP1	KDM8	NEDD9	RP11-121C2.2	RP1-99E18.2	SVILP1	ZNF887P
AC012360.6	C4BPA	CTD-2033D15.3	FSTL4	KLF3-AS1	NEK10	RP11-126O1.5	RP3-510L9.1	SYT7	ZSWIM3
AC087762.1	C4orf3	CTD-2154B17.4	GADD45B	KLHDC7A	NFATC2	RP11-134G8.10	RP4-622L5.7	TAF5L	
AC092881.1	C4orf47	CTD-2515O10.5	GAGE12H	KRT10	NPB	RP11-135A24.4	RP4-736L20.3	TAGLN	
AC114730.11	C8orf4	CTD-2535L24.2	GAL	KRT13	NPM1P9	RP11-146F11.1	RP5-1139B12.4	TAGLN2	
AC144450.2	C9orf3	CTD-2541J13.2	GAL3ST1	KRT15	NPTX1	RP11-14C10.5	RP5-890O3.9	TCAF2	
AC234582.1	CA12	CTD-2636A23.2	GALNT4	KRT18P5	NPY4R	RP11-174O3.3	RPL24P8	TCAF2P1	
ACAT2	CA5B	CTD-3065B20.2	GBE1	KRT8P3	NRIP2	RP11-17G12.2	RPL39P5	TCF7	
ACPP	CA9	CTD-3105H18.16	GBP2	KRT8P36	NUAK2	RP11-192H23.5	RPL7P23	TGFBR1	
ACTRT3	CAPN12	CTGF	GEN1	KRT9	NUDT8	RP11-196G11.6	RPS6KA5	THAP1	
ADM	CCDC137	CXCR4	GET4	LA16c-349E10.1	NUS1P1	RP11-202I11.2	RRM2	THBS1	
ADPRHL1	CCDC150P1	CYR61	GJB3	LANCL2	ODF3L1	RP11-210N13.1	RSPO3	TMEM263	
AIM1L	CCDC173	DAGLB	GMPPB	LBH	OLFML2B	RP11-213G2.1	RTN4RL2	TMEM27	
AKAP5	CCDC96	DCN	GOLGA6L7P	LCA5L	OR7E128P	RP11-213G2.2	RYR2	TMEM45A	
AKAP9	CCNE2	DERL3	GOLT1B	LCMT2	PADI1	RP11-267M23.1	S100A2	TNFRSF10D	
AL773572.7	CD3EAP	DIO2	GPR146	LDHA	PALMD	RP11-286E11.1	S100A3	TNFRSF11B	
ALDOC	CD68	DKK1	GPR75	LDHAP4	PCK1	RP11-295G20.2	SEMA7A	TNFRSF12A	
AMIGO3	CDC6	DNAH7	GPT	LINC00511	PDK3	RP11-298A10.1	SERPINB5	TNNT2	
AMOTL2	CDH5	DNAJB2	GRAMD3	LINC00680	PDSS1	RP11-29G8.3	SERPINE1	TNS4	
ANKRD1	CDK5R1	DSCAM-AS1	GRB14	LINC01260	PDXK	RP11-312J18.5	SETMAR	TONSL-AS1	
ANKRD12	CDKN2AIP	DSEL	GTF2IRD2B	LINC01341	PDZK1	RP11-332H14.2	SFMBT2	TPPP	
ANLN	CDKN2B	DUSP14	GUSBP1	LINC01468	PFKFB4	RP11-334L9.1	SFPQ	TRA2A	
AP000695.6	CEACAM7	E2F8	H19_1	LINC01588	PGAM4	RP11-347C18.5	SFR1	TRIM10	
AP001062.8	CEBPD	EDN1	HAS2	LMO7	PGM5	RP11-347E10.1	SGK1	TRIM15	
AP001469.5	CEMIP	EDN2	HBEGF	MAFB	PKD2L2	RP11-356I2.4	SGK223	TRIM53AP	
AP001626.1	CENPL	EEF1A1P16	HES4	MANF	PKDCC	RP11-362L22.1	SGO2	TRIP13	
AP1S3	CENPU	EFNA1	HES6	MAP2K3	PLAT	RP11-373N22.3	SHCBP1	TTC3-AS1	
APOBEC3A	CEP152	EFNB2	HIST1H2AE	MAP3K14	PLCH2	RP11-383J24.1	SLC12A1	TTLL11	
AREG	CFAP45	EGLN3	HIST1H2AM	MCM10	PLK2	RP11-395B7.2	SLC17A9	TTLL12	
ARFGAP1	CFAP69	EID3	HIST1H3J	MEPCE	PMEPA1	RP11-395B7.4	SLC25A1	TUBA4A	
ARHGAP33	CH17-360D5.1	EIF4HP2	HIVEP3	MFAP5	POC1B-GALNT4	RP11-399K21.11	SLC25A33	TUBAP2	
ARHGDIB	CH17-360D5.2	EME1	HMGCS1	MIAT	POFUT2	RP11-39C10.1	SLC25A45	TYMSOS	
ARID5B	CH17-360D5.3	ENTPD2	HNF4A	MICALL2	POLQ	RP11-404P21.8	SLC27A6	UBE2S	
ARL4A	CHAC2	EPS8L3	HNRNPA1P33	MIR1244-2	PPFIA4	RP1-140K8.5	SLC2A4	UHRF1	
ARNT2	CHML	ERCC6L	HNRNPA1P35	MIR222	PPP1R3C	RP11-419C5.2	SLC2A5	URB2	
ASF1B	CHRNA10	ERVV-2	HNRNPA1P4	MIR222HG	PRPS1P2	RP11-424C20.2	SLC39A10	VIPR1-AS1	
ATAD2	CIRBP-AS1	EXO5	HNRNPA1P8	MIR22HG	PSG1	RP11-435D7.3	SLC5A3	WDR37	
ATAD5	CITED2	F2R	HRASLS2	MIR635	PSG4	RP11-445H22.3	SLC7A5P2	WFIKKN1	
ATXN7L2	CKMT1B	F3	HRH1	MIR762	PSG5	RP11-449P15.2	SLCO2B1	WNT9A	
AXIN2	CLEC2B	FABP3	HSD17B7	MITF	PSPC1	RP11-45M22.2	SLFN12	WTAP	
B3GNT2	CLIC3	FAM111B	HSP90B2P	MMP7	PTGES3L	RP11-461A8.4	SLFNL1	XXyac-YM21GA2.4	
B4GALNT4	CLSPN	FAM131C	HSPA2	MPZL2	PTGS1	RP11-482M8.1	SMAD7	YPEL3	
B9D2	CMB9-22P13.1	FAM133DP	HSPA5	MSMO1	PXMP2	RP11-485O10.2	SMIM5	ZBED2	
BATF3	CNKSR3	FAM13B	HSPB3	MTCO3P12	PYCRL	RP11-490H24.5	SNORA6	ZFX	
BBOF1	COL17A1	FAM162A	HUNK	MTERF1	PZP	RP11-490O6.2	SNORD16	ZGRF1	
BCL2L15	COPRS	FAM177B	ID3	MTND1P23	RAB27B	RP11-517B11.7	SNORD2	ZNF137P	
BCL7B	CPA4	FAM222A	IDI1	MTND4P12	RAB40A	RP11-517I3.1	SNORD56	ZNF20	
BCLAF1P2	CRABP2	FAM46A	IDI2	MTND5P11	RAET1E-AS1	RP11-536P6.3	SNORD57	ZNF335	
BDKRB2	CREB3L3	FAM46B	IDI2-AS1	MT-RNR1	RARRES3	RP11-549L6.3	SNORD86	ZNF341	
BMP2	CRELD2	FAM83B	IGFBP1	MTRNR2L8	RBMXL1	RP11-624G17.3	SOX4	ZNF385A	
BMS1P7	CRIP2	FANCB	IGFL1P1	MT-TF	RCBTB2	RP11-631N16.2	SOX7	ZNF443	
BNIP3	CRISPLD2	FDFT1	IL1RAP	MTTP	RFX1	RP11-638L3.1	SOX9	ZNF485	
BORA	CRY1	FGFBP1	IL6R	MUC12	RGCC	RP11-656D10.5	SPINK13	ZNF488	
BRCA2	CRYZ	FHL1	INE2	MUC16	RGS2	RP11-65J3.14	SPINK5	ZNF503	
BRIP1	CSRNP1	FIGNL1	ING3	MUC3A	RIBC2	RP11-69I8.3	SPINK6	ZNF503-AS2	
C11orf86	CTAGE15	FIZ1	INSL4	MVD	RIF1	RP11-73M18.8	SQLE	ZNF506	

Table S4 Mass spectrometry analysis of FOXC1-interacting proteins

ABCB7	BCL2L13	CTH	GINS3	M6PR	NCLN	PFDN1	RAB2A	SMIM11A	TTC1
ABT1	BCL9L	CUTA	GIPC1	MAFK	NCOA3	PFDN4	RABGAP1	SOD2	TTC38
ACAA2	C15orf38-AP3S2	CWC27	GLRX	MALSU1	NCS1	PFKP	RBBP6	SORBS1	TUT7
ACAD9	C4BPB	DAD1	GNS	MAMLD1	NELFA	PGLS	RBM17	SPICE1	TXLNG
ACAT2	C5orf24	DBI	GOLT1B	MAOB	NFIB	PGM2	RBX1	SRCAP	TXNDC15
ACBD3	C9orf78	DCUN1D1	GOT1	MBD2	NFIC	PHC3	RELA	STIM1	TXNDC17
ACD	CAPN1	DDOST	GPN1	MCCC1	NFYA	PHF14	RETSAT	STMN1	TYSND1
ACO1	CARS1	DDT	GRN	MECP2	NNT	PIN4	RFC5	STON2	UBR4
ACOT13	CBX8	DIEXF	GSS	MED29	NOMO1	PITPNB	RGS10	STT3A	UFD1
ACTR5	CCDC9	DNAAF5	GTF2E1	MESD	NQO2	PLOD3	RHEB	STT3B	UGP2
ADK	CCNB1	DNAJB4	GTF2F2	METTL26	NSD3	PMM2	RIDA	STX8	VAT1
ADNP	CD63	DNASE2	GTF3C3	MFSD10	NSDHL	PMPCB	RNASEH2C	SUCLG2	VGLL4
ADSS2	CD9	DPP3	HARS1	MICU1	NSFL1C	PNISR	RNF13	SULT1A4	VPS29
AGFG1	CDC42EP4	DUSP12	HAT1	MIER1	NT5C2	PNP	RNF181	SUN2	VPS33A
AGO2	CDV3	DYNC1LI1	HCFC1	MLEC	NUCB1	POLB	RNPEP	SYNC	VSIR
AKAP1	CEBPG	ECI1	HIBADH	MMAB	NUCKS1	POLDIP2	RPA1	TAF10	VWA5A
AKR1A1	CENPF	EFNA1	HMBS	MRPL11	NUDT16	POLR2A	RPA3	TALDO1	WASHC4
ALDH1B1	CENPQ	EIF2B1	HMGN5	MRPL19	NUP188	POU2F1	RPP14	TBCA	WRAP53
ALDH7A1	CHAF1B	ELOA	HNF1B	MRPL28	NUP58	PPDPF	RPRD1A	TBRG4	XPO5
ANKFY1	CHAMP1	EMC1	HSDL2	MRPL32	NUP88	PPM1B	RREB1	THOC2	XPOT
ANKRD12	CHCHD2	EPS8L3	HSPBAP1	MRPL41	OAS3	PPP1R14B	RRP36	TIAL1	ZBED3
ANXA7	CHMP1A	ERC1	IBA57	MRPL53	OAT	PPP6C	SAE1	TIMM8A	ZBTB7B
ANXA8L1	CHMP4A	ERI1	IMUP	MRPS18B	OGT	PRCC	SCP2	TMX1	ZCCHC9
APOBEC3C	CHRAC1	ESS2	INIP	MRPS26	OSTC	PRIM2	SDC1	TMX2	ZFYVE19
ARAP1	CLPP	ESYT2	IRF2BPL	MSH2	PACSIN2	PRKACA	SDC4	TNRC18	ZKSCAN1
ARCN1	CNOT3	ETHE1	ISCU	MT-ATP6	PAGE1	PSMA4	SDHAF2	TNS1	ZNF280C
ARF5	COA6	FAM136A	ISOC2	MTCL1	PAPSS1	PSMB4	SEC11A	TPP1	ZNF428
ARGLU1	COLGALT1	FAM168A	KCTD12	MTHFD1L	PATJ	PSMD5	SELENOM	TRAF3IP1	ZNF593
ARHGAP1	CPOX	FAM3C	KDM2A	MVP	PCBD1	PTGES2	SERPINB6	TREX1	ZNF787
ARPP19	CPT1A	FOXC1	KLC2	NAA40	PCYT2	PTRH2	SF3A2	TRIM16	
ASF1B	CREG1	GART	KLF5	NAF1	PDIA4	PUS7	SLC1A5	TRPM8	
ATF7	CRYZ	GBE1	KNL1	NAMPT	PDIA5	PXN	SLC25A24	TSFM	
ATP5MF-PTCD1	CSNK2B	GCSH	LZIC	NAXE	PEF1	PYGL	SLC2A1	TSG101	
B4GALT5	CTBP2	GFER	LZTFL1	NCBP2	PEX19	PYURF	SLK	TST	

Table S5 Primer sets used for qRT-PCR, ChIP, and RIP

Primer set	Primers	Sequence	Product size (bp)	Application
FOXC1	Forward	5'-CCTGCCCGACTACTCTCTGCCT-3'	266	qRT-PCR
	Reverse	5'-TCCTCTGTGACTCGAACATCTC-3'		
ASNS	Forward	5'-GACAGAAGATGGATTTTTGGCT-3'	203	qRT-PCR
	Reverse	5'-CATAGAGGGCGTGCAGGGGTAC-3'		
CBS	Forward	5'-AGCAACGATGAGGAGGCGTT-3'	160	qRT-PCR
	Reverse	5'-GCACTGAGTCGGGCAGAATG-3'		
GCLM	Forward	5'-TGCACCTGCAGACGGGGAACCT-3'	150	qRT-PCR
	Reverse	5'-CTGGAAACTCCCTGACCAAATC-3'		
GPT2	Forward	5'-CCCCATCCCACAATATCCCCTC-3'	280	qRT-PCR
	Reverse	5'-GTCCTGGTACACCTCATCAGCC-3'		
FTH1	Forward	5'-TGCGCCAGAACTACCACCAGGACTC -3'	114	qRT-PCR
	Reverse	5'-CATCATCGCGGTCAAAGTAGTAAGA-3'		
ecircOGT	Forward	5'-CCCCAGAACCGTATCATTTTTT-3'	358	qRT-PCR
(divergent)	Reverse	5'-CCTGTAGGTGTCTATTGCCAGA-3'		
ecircOGT	Forward	5'-TGATGGTCGGCTGCGTGTAG-3'	290	qRT-PCR
(converget)	Reverse	5'-CATTTCGAGCGCCCTTAGTA-3'		
OGT	Forward	5'-TGATGGTCGGCTGCGTGTAG-3'	290	qRT-PCR
	Reverse	5'-CATTTCGAGCGCCCTTAGTA-3'		
EIF4A3	Forward	5'-TACCTGCCTCCAGCCACACA-3'	162	qRT-PCR
	Reverse	5'-CCTCTCCACTGCCACGAAAA-3'		
GAPDH	Forward	5'-GGGCTGCTTTTAACTCTGGT-3'	198	qPCR
	Reverse	5'-TGATTTTGGAGGGATCTCGC-3'		
U1	Forward	5'-ACTTACCTGGCAGGGGAGATACC-3'	137	qPCR
	Reverse	5'-CCACTACCACAAATTATGCAGTCG-3'		
ACTB	Forward	5'-TGCCCATCTACGAGGGGTATG-3'	156	qRT-PCR
	Reverse	5'-TCTCCTTAATGTCACGCACGATTT-3'		
ASNS	Forward	5'-GGATAGAAAGTGAGGTGGAGAAGT-3'	276	ChIP
	Reverse	5'-TGAGAGCAATGTGGGAAAGTAGGT-3'		
GPT2	Forward	5'-CCTAGTCTTGCCCTGCTCTTGCTC-3'	238	ChIP
	Reverse	5'-ACTGGCTTATGGTTTACCTCCCCC-3'		
U	Forward	5'-TGAAATCCTAACTTGCTCTG-3'	210	RIP
	Reverse	5'-TATTGTCATCATTTCGGTCC-3'		
D	Forward	5'-TAGTGTACTCTGGCTGTTGCG-3'	202	RIP
	Reverse	5'-GAGGACATCCATCCCTGTGG-3'		

ASNS, asparagine synthetase; CBS, cystathionine beta-synthase; FOXC1, forkhead box C1; GCLM, glutamate-cysteine ligase modifier subunit; GPT2, glutamic--pyruvic transaminase 2; FTH1, ferritin heavy chain 1; ecircOGT, exonic circular RNA of OGT; OGT, O-linked N-acetylglucosamine transferase; EIF4A3, eukaryotic translation initiation factor 4A3; GAPDH, glyceraldehyde 3-phosphate dehydrogenase; U1, U1 small nuclear 1; ACTB, beta-actin; U, upstream; D, downstream; RIP, RNA immunoprecipitation.

Table S6 Oligonucleotide sets used for constructs and probes

Oligo Set	Sequences
pGL3-FOXC1 Luc	5'-CGGGACGTATAAGCCGGTATAATCGCGTATACATGCGTAA-3' (Sense);<br>5'-AGCTTACGCATGTATACGCGATTATACCGGCTTATACGTCCCGGTAC-3' (Antisense)
Lenti-CV186-FOXC1 (1-1662)	5'-ATGCAGGCGCGCTACTCCGTGTCC-3' (Sense);<br>5'-TCAAAACTTGCTACAGTCGTAGAC-3' (Antisense)
Lenti-CV186-FOXC1 (S8A)	5'-CTACTCCGTGGCCAGCCCCAACTCCCTGGGAGTGGTGCCCT-3' (Sense);<br>5'-TTGGGGCTGGCCACGGAGTAGCGCGCCTGCATGGATCCCGC-3' (Antisense)
Lenti-CV186-FOXC1 (T68A)	5'-CGGGCCCTACGCGCCGCAGCCGCAGCCCAAGGACATGGTGA-3' (Sense);<br>5'-GGCTGCGGCGCGTAGGGCCCGTAGGCGCGGGCCATGCCGCC-3' (Antisense)
pCMV-3Tag-1A-FOXC1 (1-1662)	5'-CGCGGATCCATGCAGGCGCGCTACTCCGT-3' (Sense);<br>5'-CCGGAATTCTCAAAACTTGCTACAGTCGT-3' (Antisense)
pCMV-3Tag-1A-FOXC1 (1-204)	5'-CGCGGATCCATGCAGGCGCGCTACTCCGT-3' (Sense);<br>5'-CCGGAATTCCGTGTAGGGCCCGTAGGCGC-3' (Antisense)
pCMV-3Tag-1A-FOXC1 (1-642)	5'-CGCGGATCCATGCAGGCGCGCTACTCCGT-3' (Sense);<br>5'-CCGGAATTCGGGCGCGTTGCCGTCGGCCT-3' (Antisense)
pCMV-3Tag-1A-FOXC1 (205-642)	5'-CGCGGATCCCCGCAGCCGCAGCCCAAGGA-3' (Sense);<br>5'-CCGGAATTCGGGCGCGTTGCCGTCGGCCT-3' (Antisense)
pCMV-3Tag-1A-FOXC1 (205-1302)	5'-CGCGGATCCCCGCAGCCGCAGCCCAAGGA-3' (Sense);<br>5'-CCGGAATTCAGGCAGAGAGTAGTCGGGCA-3' (Antisense)
pCMV-3Tag-1A-FOXC1 (643-1302)	5'-CGCGGATCCGGTCCGCAGCCGCCGCCCGT-3' (Sense);<br>5'-CCGGAATTCAGGCAGAGAGTAGTCGGGCA-3' (Antisense)
pCMV-3Tag-1A-FOXC1 (1303-1662)	5'-CGCGGATCCCCGGTCACCAGCAGCAGCTC-3' (Sense);<br>5'-CCGGAATTCTCAAAACTTGCTACAGTCGT-3' (Antisense)
pCMV-3Tag-1A-FOXC1 Mut	5'-GCCTACGGGCGCGACACGCCGCAGCCGCAGCCCAAGGACATGG-3' (Sense)<br>5'-TGCGGCGTGTCGCGCCCGTAGGCGCGGGCCATGCCGCCCGGGT-3' (Antisense)
pMAL-c4X-FOXC1 (1-1662)	5'-CGCGGATCCATGCAGGCGCGCTACTCCGT-3' (Sense);<br>5'-CCCAAGCTTTCAAAACTTGCTACAGTCGT-3' (Antisense)
pMAL-c4X-FOXC1 (1-204)	5'-CGCGGATCCATGCAGGCGCGCTACTCCGT-3' (Sense);<br>5'-CCCAAGCTTCGTGTAGGGCCCGTAGGCGC-3' (Antisense)
pMAL-c4X-FOXC1 (1-642)	5'-CGCGGATCCATGCAGGCGCGCTACTCCGT-3' (Sense);<br>5'-CCCAAGCTTGGGCGCGTTGCCGTCGGCCT-3' (Antisense)
pMAL-c4X-FOXC1 (205-642)	5'-CGCGGATCCCCGCAGCCGCAGCCCAAGGA-3' (Sense);<br>5'-CCCAAGCTTGGGCGCGTTGCCGTCGGCCT-3' (Antisense)
pMAL-c4X-FOXC1 (205-1302)	5'-CGCGGATCCCCGCAGCCGCAGCCCAAGGA-3' (Sense);<br>5'-CCCAAGCTTAGGCAGAGAGTAGTCGGGCA-3' (Antisense)
pMAL-c4X-FOXC1 (642-1302)	5'-CGCGGATCCGGTCCGCAGCCGCCGCCCGT-3' (Sense);<br>5'-CCCAAGCTTAGGCAGAGAGTAGTCGGGCA-3' (Antisense)
pMAL-c4X-FOXC1 (1303-1662)	5'-CGCGGATCCCCGGTCACCAGCAGCAGCTC-3' (Sense);<br>5'-CCCAAGCTTTCAAAACTTGCTACAGTCGT-3' (Antisense)
Lenti-CV186-OGT (1-3141)	5'-ATGGCGTCTTCCGTGGGCAACG-3' (Sense);<br>5'-TGCTGACTCAGTGACTTCAACA-3' (Antisense)
pCMV-HA-OGT (1-3141)	5'-CGCGGTACCGGATGGCGTCTTCCGTGGGCAA-3' (Sense)<br>5'-CGCGCGGCCGCTTATGCTGACTCAGTGACTT-3' (Antisense)
pCMV-HA-OGT (TPR)	5'-CGCGGTACCGGATGGCGTCTTCCGTGGGCAA-3' (Sense)<br>5'-CGCGCGGCCGCCAGGCAATGAGCCAAGTTAC-3' (Antisense)
pCMV-HA-OGT (ΔTPR)	5'-CGCGGTACCCAGATTGTCTGTGATTGGAC-3' (Sense)<br>5'-CGCGCGGCCGCTTATGCTGACTCAGTGACTT-3' (Antisense)
pCMV-HA-OGT Mut	5'-CCCTGATGCTGACTGCAACCTAGCCAATGCTCTCAAAGAGA-3' (Sense)<br>5'-AGGTTGCAGTCAGCATCAGGGAAATGTGGTTGTAGTTCGAT-3' (Antisense)
pGEX-6P-1-OGT (1-3141)	5'-CGCGGATCCATGGCGTCTTCCGTGGGCAA-3' (Sense)<br>5'-CGCGCGGCCGCTTATGCTGACTCAGTGACTT-3' (Antisense)
pGEX-6P-1-OGT (TPR)	5'-CGCGGATCCATGGCGTCTTCCGTGGGCAA-3' (Sense)<br>5'-CGCGCGGCCGCCAGGCAATGAGCCAAGTTAC-3' (Antisense)
pGEX-6P-1-OGT (ΔTPR)	5'-CGCGGATCCCAGATTGTCTGTGATTGGAC-3' (Sense)<br>5'-CGCGCGGCCGCTTATGCTGACTCAGTGACTT-3' (Antisense)
pBiFC-FOXC1-VC155	5'-CCGGAATTCGGATGCAGGCGCGCTACTCCGTGT-3' (Sense)<br>5'-CGGCTCGAGAAACTTGCTACAGTCGTAGACG-3' (Antisense)
pBiFC-FOXC1-VC155 Mut	5'-GCCTACGGGCGCGACACGCCGCAGCCGCAGCCCAAGGACATGG-3' (Sense)<br>5'-TGCGGCGTGTCGCGCCCGTAGGCGCGGGCCATGCCGCCCGGGT-3' (Antisense)
pBiFC-OGT-VN173	5'-CGCGCGGCCGCGATGGCGTCTTCCGTGGGCAACG-3' (Sense)<br>5'-CGCGGTACCCGTGCTGACTCAGTGACTTCAACA-3' (Antisense)
pBiFC-OGT-VN173 Mut	5'-CCCTGATGCTGACTGCAACCTAGCCAATGCTCTCAAAGAGA-3' (Sense)<br>5'-AGGTTGCAGTCAGCATCAGGGAAATGTGGTTGTAGTTCGAT-3' (Antisense)

pPET28a-EGFP-FOXC1	5'-CCGGAATTCATGCAGGCGCGCTACTCCGTGT-3' (Sense)
	5'-CCCAAGCTTAAACTTGCTACAGTCGTAGACG-3' (Antisense)
pPET28a-EGFP-FOXC1 (ΔIDR)	5'-CCGGAATTCCCGCCCTATAGCTACATCGCGCTC-3' (Sense)
	5'-CCCAAGCTTCGGCACCTTGACGAAGCACTCGTT-3' (Antisense)
EGFP-N-FOXC1	5'-CCCAAGCTTATGCAGGCGCGCTACTCCGTGTCC-3' (Sense)
	5'-CGCGGATCCCCAAACTTGCTACAGTCGTAGACGAA-3' (Antisense)
EGFP-N1-FOXC1 (ΔIDR)	5'-CCCAAGCTTATGCCGCCCTATAGCTACATCGCGCTC-3' (Sense)
	5'-CGCGGATCCCCCGGCACCTTGACGAAGCACTCGTT-3' (Antisense)
pPET28a-mCherry-OGT	5'-TGGACAGCAAATGGGTCGCGGAATGGCGTCTTCCGTGGGCAA-3' (Sense)
	5'-GCCCTTGCTCACCATGGATCCTGCTGACTCAGTGACTTCAA-3' (Antisense)
pLenti-ecircOGT	5'-CTAATGACTTTTTTTTTATACTTCAGGCTGTCACCCTTGACCCAAACTTT-3' (Sense)
	5'-GGACCCCCATGGTGACTATGCCAGGTAAGAAGCAAGGAAAAGAATTAGGC-3' (Antisense)
pcDNA3.1-P570	5'-ACTATGCCAGGCTGTCACCCTGATTACAAGGATG-3' (Sense)
	5'-CAGGGTGACAGCCTGGCATAGTCACCATGGGGGT-3' (Antisense)
pGEX-6P-1-P570	5'-CGCGGATCCATGCATTATAAGGAGGCTAT-3' (Sense)
	5'-CGCGCGGCCGCTCAAGGGTGACAGCCTGGCATAGTCACCATGGGG-3' (Antisense)
ecircOGT-3FLAG	5'-GACTACAAAGATGATGACGATAAATGACCCAAACTTTCTGGATGCTTA-3' (Sense)
	5'-CTTATCGTCGTCATCCTTGTAATCAGGGTGACAGCCTGAAGTATAAAA-3' (Antisense)
ecircOGT-Mut1	5'-ACTATGCCAGCCAAGAAGCAAGGAAAAGAATTAGGCTCGGCAC-3' (Sense)
	5'-CCTTGCTTCTTGGCTGGCATAGTCACCATGGGGGTCCCTGCCC-3' (Antisense)
ecircOGT-Mut2	5'-TGACTATGCCCGGTAAGAAGCAAGGAAAAGAATTAGGCTCGG-3' (Sense)
	5'-TGCTTCTTACCGGGCATAGTCACCATGGGGGTCCCTGCCCAG-3' (Antisense)
ecircOGT-Mut3	5'-AAGCTCTGATCCATTATAAGGAGGCTATTCGAATCAGTCCTA-3' (Sense)
	5'-TCCTTATAATGGATCAGAGCTTCCTGCAGTTTTCCCTGCTGC-3' (Antisense)
ecircOGT-Mut4	5'-TGATGCATTAGAAGGAGGCTATTCGAATCAGTCCTACCTTTG-3' (Sense)
	5'-ATAGCCTCCTTCTAATGCATCAGAGCTTCCTGCAGTTTTCCC-3' (Antisense)
ecircOGT antisense probe	5'-TCAAGGGTGACAGCCTGGCATAGTCACCAT-3'
ecircOGT sense probe	5'-ATGGTGACTATGCCAGGCTGTCACCCTTGA-3'

FOXC1, forkhead box C1; OGT, O-linked N-acetylglucosamine transferase; BiFC, bimolecular fluorescence complementation; ecircOGT, exonic circular RNA of OGT.

Table S7 Oligonucleotide sets used for short hairpin RNAs and CRISPR-dCas9

Oligo Set	Sequences
sh-FOXC1 #1	5'-CCGGTGGACAGGCTGCACCTCAAGCTCGAGCTTGAGGTGCAGCCTGTCCTTTTTG-3' (Sense)
	5'-GATCCAAAAAGGACAGGCTGCACCTCAAGCTCGAGCTTGAGGTGCAGCCTGTCCA-3' (Antisense)
sh-FOXC1 #2	5'-CCGGTGACCGAGAACGGTACGTGCCTCGAGGCACGTACCGTTCTCGGTCTTTTTG-3' (Sense)
	5'-GATCCAAAAAGACCGAGAACGGTACGTGCCTCGAGGCACGTACCGTTCTCGGTCA-3' (Antisense)
sh-OGT #1	5'-CCGGTGCTGAGCAGTATTCCGAGAAACTCGAGTTTCTCGGAATACTGCTCAGCTTTTTG-3' (Sense)
	5'-GATCCAAAAAGCTGAGCAGTATTCCGAGAAACTCGAGTTTCTCGGAATACTGCTCAGCA-3' (Antisense)
sh-OGT #2	5'-CCGGTTTTAGCACTCTGGCAATTAAACTCGAGTTTAATTGCCAGAGTGCTAAATTTTTG-3' (Sense)
	5'-GATCCAAAAATTTAGCACTCTGGCAATTAAACTCGAGTTTAATTGCCAGAGTGCTAAAA-3' (Antisense)
sh-EIF4A3 #1	5’-CCGGTGCTCTCGGTGACTACATGAATCTCGAGATTCATGTAGTCACCGAGAGCTTTTTG-3’ (Sense)
	5’-GATCCAAAAAGCTCTCGGTGACTACATGAATCTCGAGATTCATGTAGTCACCGAGAGCA-3’ (Antisense)
sh-EIF4A3 #2	5’-CCGGTGCAATCCAGCAACGAGCAATCCTCGAGGATTGCTCGTTGCTGGATTGCTTTTTG-3’ (Sense)
	5’-GATCCAAAAAGCAATCCAGCAACGAGCAATCCTCGAGGATTGCTCGTTGCTGGATTGCA-3’ (Antisense)

FOXC1, forkhead box C1; OGT, O-linked N-acetylglucosamine transferase; EIF4A3, eukaryotic translation initiation factor 4A3.
